# A Max-Flow Approach to Random Tensor Networks

**DOI:** 10.3390/e27070756

**Published:** 2025-07-15

**Authors:** Khurshed Fitter, Faedi Loulidi, Ion Nechita

**Affiliations:** 1 Quantum Science and Engineering Department, Ecole Polytechnique Federal de Lausane, 1015 Lausanne, Switzerland; khurshed.fitter@epfl.ch; 2 Networked Quantum Devices Unit, Okinawa Institute of Science and Technology Graduate University, Onna-son 904-0495, Okinawa, Japan; faedi.loulidi@oist.jp; 3 Laboratoire de Physique Théorique, Université de Toulouse, CNRS, UPS, 31062 Toulouse, France

**Keywords:** random tensor networks (RTN), entanglement entropy, max-flow min-cut theorem, free probability theory, AdS/CFT correspondence, Ryu–Takayanagi formula, series-parallel graphs, free convolution, Marchenko–Pastur distribution

## Abstract

The entanglement entropy of a random tensor network (RTN) is studied using tools from free probability theory. Random tensor networks are simple toy models that help in understanding the entanglement behavior of a boundary region in the anti-de Sitter/conformal field theory (AdS/CFT) context. These can be regarded as specific probabilistic models for tensors with particular geometry dictated by a graph (or network) structure. First, we introduce a model of RTN obtained by contracting maximally entangled states (corresponding to the edges of the graph) on the tensor product of Gaussian tensors (corresponding to the vertices of the graph). The entanglement spectrum of the resulting random state is analyzed along a given bipartition of the local Hilbert spaces. The limiting eigenvalue distribution of the reduced density operator of the RTN state is provided in the limit of large local dimension. This limiting value is described through a maximum flow optimization problem in a new graph corresponding to the geometry of the RTN and the given bipartition. In the case of series-parallel graphs, an explicit formula for the limiting eigenvalue distribution is provided using classical and free multiplicative convolutions. The physical implications of these results are discussed, allowing the analysis to move beyond the semiclassical regime without any cut assumption, specifically in terms of finite corrections to the average entanglement entropy of the RTN.

## 1. Introduction

The AdS/CFT correspondence is described as a quantum theory (more precisely, a *conformal field theory*) lying on the boundary of an *anti-de Sitter* spacetime geometry [1]. Many particular features of this correspondence remain mysterious, in particular the link with quantum information theory and *entanglement*. It was shown in [2] that for a fixed time slice, the entanglement behavior of a given region of the boundary quantum theory is proportional to the minimal hypersurface bulk area homologous to the region of interest (known as the Ryu–Takayanagi entanglement entropy). In the context of AdS/CFT, the Ryu-Takayanagi formula demonstrates a crucial link between the entanglement behavior of an intrinsic quantum theory and its connection with the bulk gravitational field. These results open up a new perspective on the understanding of quantum gravity in the AdS/CFT framework from the viewpoint of entanglement and quantum information theory. For a complete introduction, see [3] and references therein.

The difficulty of computing the entanglement properties of boundary quantum theories has led to the development of attractable simple models, particularly the *tensor network* and *random tensor network* frameworks. Initially, the tensor network framework started with “good” models approximating ground states in condensed matter physics. In the context of condensed matter physics, tensor networks represent ground states of a class of gapped Hamiltonians [4]. Moreover, tensor networks have paved the way to understanding different physical properties such as the classification of topological phases of matter. An extensive review of all the different applications can be found in [4]. Recently, other extensions of tensor networks to random tensor networks for studying random matrix product states or projected entangled pairs of states have been introduced in [5,6,7]. The random tensor network (or simply RTN) was initiated in [8] as a toy model reproducing the key properties of entanglement behavior in the AdS/CFT context [9,10,11,12,13,14,15]. Moreover, the random tensor network framework has appeared in different active areas of research ranging from condensed matter physics to random quantum circuits and measurement frameworks [16,17,18,19,20,21,22,23,24].

In general, a random tensor network (or simply RTN) is defined as a random quantum state via a given fixed graph structure, as will be described below. The main problem is to compute the *average entanglement* between two complementary regions of the graph as D→∞, where *D* denotes the dimension of the Hilbert space of the model. Various results have been established, allowing the entanglement entropy of RTN models to be understood as toy models that mimic the entanglement behavior in quantum gravity. The scaling of the entanglement entropy (in the D→∞ limit) as a function of the size and *number of minimal cuts* needed to separate the region of interest from the rest of the graph has been explored in different works [8,25]. Moreover, several directions have been explored that move beyond the toy model picture of (random) tensor networks [26,27].

The focus of this work is a *maximal flow* approach to the analysis of general random tensor networks. The use of the maximal flow approach was already explored in [10] to compute the entanglement negativity, in [28] to derive the Ryu–Takayanagi entanglement entropy in the continuum setting, and in Appendix C of [8] in a moment computation similar to the ones discussed in this work; see also [9,25]. As described in the previous paragraph, the model consists of defining a random quantum state from a given fixed graph structure. This model consider a graph with edges (bulk edges) and half-edges (boundary edges). The use of bulk and boundary edges will become clear from the definition of the model. A finite-dimensional Hilbert space $C^{D}$ is associated with each half-edge and a tensor product Hilbert space ${\left(C^{D}\right)}^{⊗2}$ with each edge. The edge Hilbert spaces generate a local Hilbert space associated with each vertex of the graph. In order to define an RTN, each component of the graph is associated with a quantum state generated at random. For this, a random Gaussian state is generated for each vertex and a *maximally entangled state* is associated with each of the edges. A *random tensor network* is defined by projecting all of the maximally entangled states associated with the graph onto the total random states generated in each vertex. The obtained random tensor network lies in the full-boundary Hilbert space. The main goal of this work is to consider a sub-boundary region *A* of the graph and evaluate the entanglement behavior of the associated residual state as D→∞. The first computation is to estimate the moment computation of the state associated with the region *A* as D→∞. With the help of the *maximal flow*, which is developed in detail in this work, the moments are estimated *without any assumption regarding cuts*, and it is shown that they converge to the moment of a graph-dependent measure. It is shown that if the obtained *partial order* is *series-parallel*, the measure associated with the graph can be explicitly constructed *without any cut assumption* through the use of *free probability theory*. Moreover, the *existence of higher-order correction terms* of the entanglement entropy (provided by a graph-dependent measure) is demonstrated, and these can be explicitly provided if the partial order is series-parallel. In different examples, it is shown how the measures associated with the initial graph can be computed explicitly in the case where the obtained partial order is series-parallel. The link between quantum information theory, free probability, and random tensor networks was already explored in [25]. There, a general link state was used to represent the effect of the bulk matter field in the AdS/CFT context, extending the semiclassical regime with correction terms of the entanglement structure. Moreover, the obtained results in [25] *assumed* the existence of two disjoint minimal cuts separating region *A* from the rest. In this work, only maximally entangled states in the bulk edge of the model are used; higher-order correction terms are obtained without any cut assumption, which may be interpreted as *intrinsic fluctuations*. In the context of AdS/CFT, these are intrinsic to the quantum spacetime nature of bulk gravitational field without any bulk matter field.

The main differences between this work and the previous literature [8,25] are highlighted below. Through the use of the maximum flow approach, we emphasize the importance of the partial order defined by disjoint paths achieving the maximum flow to the computation of the correction term for the entropy of entanglement of the random tensor network states. Indeed, both the minimum cut and the maximum flow perspectives provide the dominating term (∼logD). However, performing the analysis with the maximum flow allows for a combinatorial characterization of the moments of the reduced state. Moreover, in the case where the flow partial order is *series-parallel*, an explicit formula for the correction term is provided in terms of a probability measure constructed from the network structure using both the free and classical multiplicative convolutions from (free) probability theory.

The rest of this work is organized as follows. Section 2 provides a summary of the main results. Section 3 introduces the random tensor network framework. Section 4 presents the moment computation of a given state $\rho_{A}$ associated with a given sub-boundary region of the graph *A*. Section 5 uses the maximal flow approach to compute the asymptotic scaling of the moments and demonstrates convergence to a graph-dependent measure. Section 6 introduces the notion of series-parallel partial order and uses the free probability to explicitly show how a graph-dependent measure can be constructed with the free product convolution and classical measurement product. Section 7 provides various examples of random tensor networks and explicitly shows the associated measure obtained in the case of an obtained partial order that is series-parallel. Section 8 presents the main technical results, which use concentration inequalities to show that the obtained higher-order entanglement correction terms are graph-dependent without any cut assumption; moreover, the graph-dependent measure can be explicitly constructed if the partial order is series-parallel.

## 2. Main Results

This section introduces the main definitions and results obtained in this work. The focus is placed on the computation of the entanglement entropy of a given random tensor network. The most general framework of random tensor networks is considered, and the entanglement structure of the random tensor is studied with respect to a fixed bipartition of the total Hilbert space. By addressing the problem through a *network flow approach*, the leading term can be computed along with the higher-order correction terms of the entanglement entropy, which are *graph-dependent*. The higher-order correction terms are found to play a crucial role in various areas, particularly in the context of AdS/CFT [8,25,27,29], as will be discussed following the presentation of the main results. The key results of this work can be informally summarized as follows.

In the limit of large local Hilbert space dimension *D*, the average Rényi entanglement entropy of an RTN *G* across a given bipartition (A|B) has the following:

-   A dominating term of the form $\mathrm{maxflow}(G_{A|B})\cdot \log D$
-   A finite correction term which is graph-dependent

In the case where $G_{A|B}$ is a *series-parallel* graph, the distribution of the entanglement spectrum (and consequently the finite entropy correction) can be computed as an iterative classical and free convolution of Marc̆henko–Pastur distributions.

**Figure 1:**
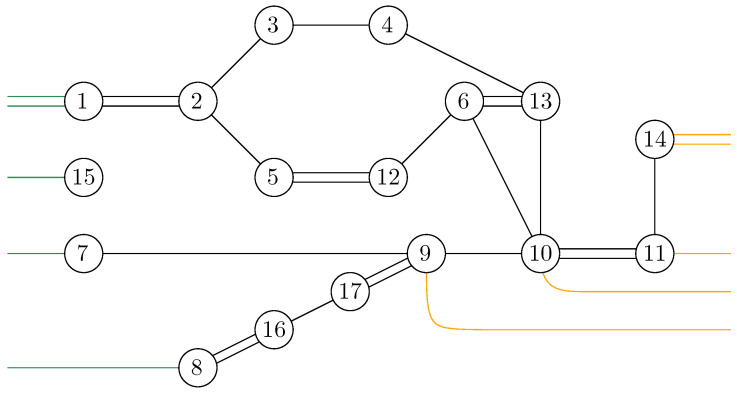
A tensor network depicting a tensor from ${\left(C^{D}\right)}^{⊗10}$ obtained by contracting 17 tensors. The ten Hilbert space factors are partitioned into two subsets *B* ⊔ *A*.

A random tensor network is associated with a corresponding random quantum state $|\psi_{G}\rangle$ that encodes the structure of a graph *G*. To describe this, the graph *G* and relevant terminology are introduced below. Further technical details and definitions of the model are provided in Section 3. Let G=(V,E) denote a connected undirected finite graph with (full) edges and half-edges (sometimes referred to as an *open graph*); the former encode the internal entanglement structure of the quantum state $|\psi_{G}\rangle$, while the latter represent the physical systems (Hilbert spaces) on which $|\psi_{G}\rangle$ is supported. The sets of edges (bulk edges) and half-edges (boundary edges) are denoted by $E_{b}$ and $E_{\partial }$, respectively. Formally, the sets of edges and half-edges are provided by $E_{b}:=\left\{e_{x,y}\;|\;e_{x,y}=\left(x,y\right):\;x,y\in V\right\}$ and $E_{\partial }:=\left\{e_{x}=\left(x,\cdot \right):\;x\in V\right\}$, where $E:=E_{b}⊔E_{\partial }$. The corresponding *random tensor* $|\psi_{G}\rangle$ is then defined as

$$|\psi_{G}\rangle :=\langle \underset{e\in E_{b}}{⨂}\Omega_{e}\;|\;\underset{x\in V}{⨂}g_{x}\rangle \in {\left(C^{D}\right)}^{⊗|E_{\partial }|},$$

where $|g_{x}\rangle$ are taken to be random Gaussian states defined in the local Hilbert space of each vertex *x*. Moreover, a maximally entangled state $|\Omega_{e}\rangle \in C^{D}⊗C^{D}$ is associated with each (full) edge $e\in E_{b}$, which serves to contract the internal degrees of freedom of the tensor network. A representation of a random tensor network is provided in Figure 1, which is discussed in detail to illustrate the main results of this work. Further details are provided in Definition 3.

As mentioned earlier, the objective of this work is to evaluate the *entanglement entropy* of the random quantum state $|\psi_{G}\rangle$ along a bipartition A|B of the boundary edges $E_{\partial }=A⊔B$. This analysis is carried out in the limit of the large local Hilbert space dimension D→∞. To evaluate the entanglement entropy of the pure state $|\psi_{G}\rangle$, the asymptotic *entanglement spectrum* along the bipartition A|B is computed, that is, the limiting spectrum of the density matrix $\rho_{A}={\mathrm{Tr}}_{B}|\psi_{G}\rangle \langle \psi_{G}|$. From this spectral information, the average Rényi entanglement and von Neumann entropies for the approximate normalized state ${\tilde{\rho }}_{A}$ can be deduced, respectively provided by:

$${\tilde{\rho }}_{A}:=D^{-|E_{\partial }|}\rho_{A}\to \left\{\begin{array}{cc} \lim_{D\to \infty }E\;S_{n}\left({\tilde{\rho }}_{A}\right)\;\mathrm{with}\;S_{n}\left(\rho \right):=\frac{1}{1-n}\log\left(\mathrm{Tr}\rho^{n}\right), & \\ \lim_{D\to \infty }E\;S\left({\tilde{\rho }}_{A}\right)\;\mathrm{with}\;S\left(\rho \right):=-\mathrm{Tr}\left(\rho \log\rho \right). \end{array}\right.$$

Above, the expectation is taken with respect to the Gaussian distribution of the independent random tensors $|g_{x}\rangle$ present at each vertex of the graph. The use of approximate normalized state instead of a correctly normalized state $\rho_{A}/\mathrm{Tr}\rho_{A}$ will become clear from Section 8.

First, the *moments* of the random matrix $\rho_{A}$ are computed exactly, then the main contributing terms at large dimensions are analyzed by relating the problem to a *maximum flow* question in a related graph. By employing the maximal flow and tools from *free probability theory*, the leading and fluctuating terms of the Rényi entropy are derived and the behavior of the von Neumann entanglement entropy is subsequently deduced.

**Moment computation.** First, the un-normalized state $\rho_{A}$ is considered and its moments are computed. As a first step, the *graphical Wick formula* from [30] is used to obtain

(1) $$E\left[\mathrm{Tr}(\rho_{A}^{n})\right]=\sum_{\alpha =\left(\alpha_{x}\right)\in S_{n}^{\left|V\right|}}D^{n|E_{\partial }|-H_{G}^{\left(n\right)}\left(\alpha \right)},\;\forall \;n\in N,$$

where $H_{G}^{\left(n\right)}\left(\alpha \right)$ can be understood as the Hamiltonian of a classical “spin system” in which each spin variable takes a value from the permutation group $S_{n}$:

$$H_{G}^{\left(n\right)}\left(\alpha \right):=\sum_{(x,\cdot )\in A}|\gamma_{x}^{-1}\alpha_{x}|+\sum_{(x,\cdot )\in B}|{\mathrm{id}}_{x}^{-1}\alpha_{x}|+\sum_{\left(x,y\right)\in E_{b}}\left|\alpha_{x}^{-1}\alpha_{y}\right|.$$

In the above, the identity permutation ${\mathrm{id}}_{x}\in S_{n}$ is associated with the region *B* (corresponding to taking the partial trace over *B*), while the full-cycle permutation $\gamma_{x}=\left(n\;n-1\;\ldots \;2\;1\right)$ is associated with the region *A* (corresponding to the trace of the *n*-th power of $\rho_{A}$). A more precise statement and proof can be found in Proposition 1 in Section 4. It should also be noted that the contribution of the normalization term of ${\tilde{\rho }}_{A}$ is provided by

$$E\left[{\left(\mathrm{Tr}\rho_{A}\right)}^{n}\right]=\sum_{\alpha =\left(\alpha_{x}\right)\in S_{n}^{\left|V\right|}}D^{n|E_{\partial }|-h_{G}^{\left(n\right)}\left(\alpha \right)},\;\forall \;n\in N$$

where

$$h_{G}^{\left(n\right)}\left(\alpha \right):=\sum_{\left(x,\cdot \right)\in E_{\partial }}|{\mathrm{id}}_{x}^{-1}\alpha_{x}|+\sum_{\left(x,y\right)\in E_{b}}\left|\alpha_{x}^{-1}\alpha_{y}\right|.$$

Note that in the above formula the quantity $h_{G}^{\left(n\right)}\left(\alpha \right)$ is simply $H_{G}^{\left(n\right)}\left(\alpha \right)$ with A=∅; see Proposition 2 for more details. Note that in the particular case with n=2, the authors of [8] provided an exact mapping to the partition function of a classical Ising model. Notice the frustrated boundary conditions of the Hamiltonian above; vertices connected to region *A* prefer the configuration $\alpha_{x}=\gamma_{x}$, while vertices connected to region *B* prefer the low energy state $\alpha_{x}={\mathrm{id}}_{x}$. The connection between the combinatorics of the dominating terms in the random matrix model considered here and an effective spin system Hamiltonian have already been extensively studied in the quantum statistical mechanics literature; see, e.g., [22,31,32].

**Maximal flow.** The *(max)-flow* approach consists of identifying the leading terms from the moment formula above as D→∞. For this, a *network* $G_{A|B}$ is introduced. This network is derived from the original graph *G* by connecting all of the half-edges in *A* to an extra vertex γ (sink) and all of the half-edges in *B* to id (source). In $G_{A|B}$, the vertices are valued in the permutation group $S_{n}$, with all the half-edges connected either to the source id or to the sink γ. The flow approach consists of looking at the different paths starting from the source id to the sink γ. The different paths in the flow approach induce an ordering structure (more precisely a *poset structure*) in the network $G_{A|B}$. Intuitively, the maximal flow will consist of searching the maximal number of these paths such that the source and sink will be disconnected upon removing the paths from the network. More precisely, by *Menger’s theorem*, the maximum flow in this graph is equal to the number of edge-disjoint *augmenting paths* that start from the source id and end in the sink γ. Figure 7 represents the different paths achieving maximal flow in the network $G_{A|B}$ from the original graph *G* as represented in Figure 1. This procedure allows us to find a *lower bound* to the Hamiltonian $H_{G_{A|B}}^{\left(n\right)}\left(\alpha \right)$ that can be attained by some choice of the variables $\alpha_{x}$.

**Theorem** **1.**  *For all n≥1, one has*

$$\min_{\alpha \in S_{n}^{\left|V\right|}}H_{G_{A|B}}^{\left(n\right)}\left(\alpha \right)=\left(n-1\right)\mathrm{maxflow}\left(G_{A|B}\right),$$

*where $H_{G_{A|B}}^{\left(n\right)}\left(\alpha \right)$ denotes the extended Hamiltonian in the network $G_{A|B}$. After all augmenting paths achieving the maximum flow in $G_{A|B}$ have been removed, a* clustered graph *$G_{A|B}^{c}$ remains, which is formed by clustering all the remaining connected components (see Figure 8). Importantly, the maximality of the flow implies that the cluster-vertices [id] and [γ] in this clustered graph are disjoint. Further details and the proof of the above result are provided in Proposition 5.*

As a direct consequence of the above result, the moment convergence as D→∞ can be deduced; further details of the following result are provided in Theorem 4.

**Theorem** **2.**  *In the limit D→∞, for all n≥1 one has*

$$\lim_{D\to \infty }E\frac{1}{D^{F(G_{A|B})}}\left[\mathrm{Tr}\left({\left(D^{F\left(G_{A|B}\right)-\left|E_{\partial }\right|}\;\rho_{A}\right)}^{n}\right)\right]=m_{n},$$

*where $m_{n}$ are the moments of a probability measure $\mu_{G_{A|B}}$ and $F\left(G_{A|B}\right)=\mathrm{maxflow}\left(G_{A|B}\right)$.*

Moreover, we can show that the normalization term converges to 1, as shown in Corollary 1. The previous maximum flow computation provides the first order in the formula for the average entanglement entropy of random tensor network states:

$$E\left[S_{n}\left({\tilde{\rho }}_{A}\right)\right]\approx \mathrm{maxflow}\left(G_{A|B}\right)\cdot \log D\;\forall \;n\geq 1.$$

**Free probability theory and entanglement.** The main contribution of this work is to show that the *second order* (or the finite corrections) of the Rényi and von Neumann entanglement entropy can be found by carefully analyzing the set of augmenting paths achieving the maximum flow in the graph $G_{A|B}$. When the different paths achieve the maximal flow in the graph $G_{A|B}$, a *partial order* $G_{A|B}^{o}$ is obtained after the clustering operation, where the vertices are the different permutation clusters formed from the clustered graph $G_{A|B}^{c}$. Figure 9 shows the obtained partial order from the original graph *G* in Figure 1. The results are general, and become explicit in the setting where the partial order $G_{A|B}^{o}$ is *series-parallel*. With the help of *free probability theory*, the second-order correction terms of each of the Rényi and von Neumann entropy can be deduced in this setting.

**Definition** **1.**  *A graph G is called* series-parallel *if it can be constructed recursively using the following two operations:*

- *Series concatenation:* G=H1SH2 *is obtained by identifying the sink of* $H_{1}$ *with the source of* $H_{2}$*.*
- *Parallel concatenation:* G=H1PH2 *obtained by identifying the sources and sinks of* $H_{1}$ *and* $H_{2}$*.*

**Definition** **2.**  *A probability measure $\mu_{G}$ is associated with a series-parallel graph G, defined recursively as follows:*

- *The Dirac mass at* 1 *is associated with the trivial graph $G_{\mathrm{triv}}=\left(\left\{s,t\right\},\left\{\left\{s,t\right\}\right\}\right)$, as follows: $\mu_{G_{\mathrm{triv}}}:=\delta_{1}$.*
- *Series concatenation: μGSH:=μG⊠MP⊠μH. Here*
$$d\mathrm{MP}:=\frac{1}{2\pi }\sqrt{4t^{-1}-1}\;dt$$
  *is the Marc̆henko–Pastur distribution and* ⊠ *is the free convolution product. For more details, see Appendix A.*
- *Parallel concatenation: μGPH:=μG×μH.*

**Theorem** **3.**  *In the limit D→∞, the average Rényi entanglement entropy ∀n≥1 and von Neumann entropy of an approximate normalized state ${\tilde{\rho }}_{A}:=D^{-|E_{\partial }|}\rho_{A}$ respectively behave as follows:*

$$\begin{array}{cc} E\left[S_{n}\left({\tilde{\rho }}_{A}\right)\right] & =\mathrm{maxflow}\left(G_{A|B}\right)\cdot \log D-\frac{1}{n-1}\log\int t^{n}\;d\mu_{G_{A|B}}\left(t\right)+o\left(1\right),\\ E\left[S({\tilde{\rho }}_{A})\right] & =\mathrm{maxflow}\left(G_{A|B}\right)\cdot \log D-\int t\;\log t\;d\mu_{G_{A|B}}+o\left(1\right). \end{array}$$

For further details and the proof of the above statements, the reader is referred to Corollary 2. In particular, if the obtained partial order $G_{A|B}^{o}$ is series-parallel, then the measure $\mu_{G_{A|B}}=\mu_{G_{A|B}^{o}}$ can be explicitly constructed, as detailed in Theorem 5. The use of the approximate normalized state rather than the normalized state ${\tilde{\rho }}_{A}:=\rho_{A}/\mathrm{Tr}\rho_{A}$ is justified by the concentration result for $\mathrm{Tr}\rho_{A}$ presented in Section 8.1.

It was previously argued in [8,27] that if one wants to encode the *quantum fluctuations*, then instead of a maximally entangled state it is necessary to use a general “link state” $|\varphi_{e}\rangle$ defined by

$$e\in E_{b}\to |\varphi_{e}\rangle :=\sum_{i=1}^{D}\sqrt{\lambda_{e,i}}|i_{x},i_{y}\rangle .$$

It was recently shown in [25] that the quantum fluctuations beyond the semiclassical regime in AdS/CFT are obtained for the non-flat spectra of the link state under the existence *assumption* of *two minimal cuts*. The use of a generic link state in the context of AdS/CFT represents the bulk matter field contribution.In this work, the *maximal flow* approach has been used to demonstrate the existence of quantum fluctuations without any *minimal cut assumption* and with the *maximally entangled state* serving as the link state. The higher-order correction terms obtained in this context can be interpreted as the “intrinsic” quantum fluctuations of spacetime geometry in the absence of any bulk matter field represented by a general link state.

For example, in the case of the graph represented in Figure 1, the resulting partial order $G_{A|B}^{o}$ is series-parallel (see Figure 9), where

GA|Bo=G1SG2SG3withμGA|Bo=μG1⊠MP⊠μG2⊠MP⊠μG3=μG1⊠MP⊠2.

As represented in Figure 10, the graphs $G_{2}$ and $G_{3}$ are trivial; hence, $\mu_{G_{2}}=\mu_{G_{3}}=\delta_{1}$. The graph $G_{1}$ can be factored as a parallel composition of two other graphs, as represented in Figure 11:

G1=G5PG4withμG1=μG4×μG5.

The graph $G_{4}$, as represented in Figure 12, factorizes as follows:

G4=G6PG7SG8withμG4=μG6×μG7⊠MP⊠μG8=MP×MP⊠MP

where we use the fact that $G_{6}$ and $G_{7}$ are series compositions of two trivial graphs; thus, $\mu_{G_{6}}=\mu_{G_{7}}=\mathrm{MP}$, while $\mu_{G_{8}}=\delta_{1}$.

Moreover, the graph $G_{5}$, as represented in Figure 13, factorizes as follows:

G5=G9SG10SG11PG12SG13

with the associated measure

$$\mu_{G_{5}}=\mu_{G_{9}}⊠\mathrm{MP}⊠\mu_{G_{10}}⊠\mathrm{MP}⊠\left(\mu_{G_{11}}\times \mu_{G_{12}}\right)⊠\mathrm{MP}⊠\mu_{G_{13}}={\mathrm{MP}}^{⊠3}⊠\left({\mathrm{MP}}^{⊠2}\times \mathrm{MP}\right),$$

where the series composition for $G_{11}$ and $G_{12}$ has been used iteratively, with their respective measures provided by $\mu_{G_{11}}={\mathrm{MP}}^{⊠2}$ and $\mu_{G_{12}}=\mathrm{MP}$. In the case of random tensor network represented in Figure 1, the partial order is series-parallel, with the associated measure

GA|Bo=G1SG2SG3,

with

$$\mu_{G_{A|B}^{o}}=\left\{\left[{\mathrm{MP}}^{⊠3}⊠\left({\mathrm{MP}}^{⊠2}\times \mathrm{MP}\right)\right]\times \left[(\mathrm{MP}\times \mathrm{MP})⊠\mathrm{MP}\right]\right\}⊠{\mathrm{MP}}^{⊠2},$$

which is obtained by combining all the results stated above. The minimal cuts associated with the network $G_{A|B}$ (see Figure 7) can also be considered, as represented in Figure 14. It should be noted that several minimal cuts exist; for simplicity, four ways of achieving the minimal cut in the network are represented. Note that some of the cuts share common edges.

## 3. Random Tensor Networks

In this section, starting from a given (open) graph with edges (bulk edges) and half-edges (boundary edges), the random tensor network model is introduced. For this purpose, a Hilbert space is associated with each edge and half-edge of the graph. The edge Hilbert spaces induce a local Hilbert space for each vertex in the graph. A random Gaussian state is assigned to each of the vertices, and a maximally entangled state is associated with each edge. The random tensor network is defined by projecting all of the maximally entangled states associated with the edges of the graph onto the vertex states, which are provided by the tensor product of all the random Gaussian vectors. The main definitions of the model are introduced in this section, and the different entanglement notions are recalled as well.

Section 3.1 introduces the random tensor network model, after which Section 3.2 recalls the different entanglement notions and their properties.

### 3.1. Random Tensor Network

The construction of the random tensor network model is presented in the following. Let G=(V,E) be a bulk connected undirected finite open graph with edges and half-edges. The multi-sets of edges and half-edges are denoted by $E_{b}$ and $E_{\partial }$, respectively; multiple edges are allowed in the graph. Formally, bulk edges $e\in E_{b}$ are of the form e=(x,y) for some x,y∈V, while half-edges $e^{\prime }\in E_{\partial }$ are of the form $e^{\prime }=\left(x,\cdot \right)$ for some x∈V, where the · indicates that $e^{\prime }$ is a partial edge. The total set of edges *E* is decomposed as

$$E:=E_{b}⊔E_{\partial }.$$

Bulk connectivity is assumed, meaning that all vertices in the bulk region of the graph are connected; this corresponds to the “connected network” property as defined in Definition 2 of [33]. The cardinalities of the bulk, boundary, and total edge multi-sets are denoted by $|E_{b}|$, $|E_{\partial }|$, and $\left|E|=\right|E_{b}\left|+\right|E_{\partial }|$, respectively.

A Hilbert space $C^{D}$ is associated with each half-edge on a given vertex in the graph, while the Hilbert space $C^{D}⊗C^{D}$ is assigned to each bulk edge connecting two vertices, where *D* is a finite parameter known as the *bond dimension*. A random Gaussian vector is defined for each vertex of the graph state, lying in the local Hilbert space associated with that vertex. Furthermore, a maximally entangled state is associated with each edge of the graph. Thus, the *random tensor network* is defined as a random quantum state constructed by projecting the total tensor product of the random Gaussian states for each vertex onto all of the maximally entangled states formed along the bulk edges (see Definition 3).

Formally, Hilbert spaces are associated with each part of the graph *G* as follows:

- A finite-dimensional Hilbert space $H_{e_{x}}$ is assigned to each half-edge defined on a vertex *x*:
  $$\begin{array}{cc} e_{x}\in E_{\partial } & \to H_{e_{x}}:=C^{D}\\ E_{\partial } & \to H_{\partial }:=\underset{e_{x}\in E_{\partial }}{⨂}H_{e_{x}}. \end{array}$$
- A Hilbert space $H_{e_{x,y}}$ is associated with each edge $e_{x,y}\in E_{b}$:
  $$e_{x,y}\in E_{b}\to H_{e_{x,y}}=H_{e\to x}⊗H_{e\to y}:=C^{D}⊗C^{D},$$
  where $H_{e_{x,y}}$ denotes the Hilbert space connecting the two vertices *x* and *y*, which is composed as a tensor product of the two corresponding half-edges.
- For each vertex x∈V, the local vertex Hilbert space $H_{x}$ is defined as follows:
  $$\begin{array}{cc} x\in V & \to H_{x}:=\underset{E∋e\to x}{⨂}H_{e\to x}\\ V & \to H_{V}:=\underset{x\in V}{⨂}H_{x}=\underset{x\in V}{⨂}\underset{E∋e\to x}{⨂}H_{e\to x}. \end{array}$$
  Above, the Hilbert space $H_{x}$ represents the local Hilbert space associated with a vertex *x* defined as all the edges of Hilbert space that contribute locally; the notation e→x denotes that the edge *e* is incident to the vertex *x*.

Having defined the general Hilbert space structure associated with a generic graph *G*, in the following we define quantum states in the graph *G* in order to introduce the random tensor network model. Each vertex x∈V is associated with a random vector (un-normalized quantum state) $|g_{x}\rangle \in H_{x}$ sampled from an *i.i.d. Gaussian distribution*:

$$\begin{array}{cc} x\in V & \to |g_{x}\rangle \in H_{x}\\ V & \to \underset{x\in V}{⨂}|g_{x}\rangle \in H_{V}. \end{array}$$

A normalized maximally entangled state $|\Omega_{e}\rangle$ is also associated with each bulk edge $e_{x,y}\in E_{b}$, provided by

(2) $$\begin{array}{cc} e_{x,y}\in E_{b} & \to H_{e_{x,y}}\\ e_{x,y} & \to |\Omega_{e}\rangle :=\frac{1}{\sqrt{D}}\sum_{i=1}^{D}|i_{x},i_{y}\rangle , \end{array}$$

where $|i_{x}\rangle$ and $|i_{y}\rangle$ indicate the state associated with the vertex *x* sharing an edge with *y*.

**Definition** **3.**  *A* random tensor network *$|\psi_{G}\rangle$ is defined as a projection of the vertex state over all of the maximally entangled states $|\Omega_{e}\rangle$ for each $e_{x,y}$ in $E_{b}$, where*

(3) $$|\psi_{G}\rangle :=\langle \underset{e\in E_{b}}{⨂}\Omega_{e}\;|\;\underset{x\in V}{⨂}g_{x}\rangle \in {\left(C^{D}\right)}^{⊗|E_{\partial }|}.$$

It should be mentioned that the following example is used in all other parts of this work as an illustration of the different results obtained in each section.

**Example** **1.**  *An illustration of a random tensor network is provided in Figure 1, where the boundary region of G is provided by all the half-edges $E_{\partial }:=\left\{e_{1},e_{15},e_{7},e_{8},e_{9},e_{10},e_{11},e_{14}\right\}$. In Figure 1, the region A is defined as the set of half-edges at the vertices {9,10,11,14}, that is, $A:=\{e_{9},e_{10},e_{11},e_{14}\}$. The complementary region $B:=E_{\partial }\setminus A$ consists of the half-edges associated with the vertices {1,15,7,8}, namely, $B:=\{e_{1},e_{15},e_{7},e_{8}\}$.*

It should also be mentioned that in the present construction of the random tensor network, the edges and half-edges are used to generate the vertex Hilbert space $H_{x}$. Other types of random tensor network models have already been explored in the literature; see [8,9,25] and the references therein. In the previously mentioned models, the bulk and boundary vertices are first defined, whereas in the present approach the focus is placed on the edges and half-edges, which generate the local Hilbert space for each vertex, with the bulk states provided by a maximally entangled state. The initial work on random tensor networks was presented in [8], where the aim was to compute the entanglement entropy of a subregion of the random tensor network; as the bond dimension tends to infinity, this becomes proportional to the minimal cuts of the graph, reproducing the famous Ryu–Takayanagi entanglement entropy [2] in a discrete version.

In a recent work [25], the authors associated a state with a general “link” state connecting two bulk vertices, thereby generalizing previous models in which the existence of two non-crossing minimal cuts was allowed. This result allowed them to compute higher-order correction terms of the entanglement entropy. In this work, our main goal is to derive the higher-order correction terms using the maximal flow approach without any minimal cut assumption, with maximally entangled states connecting the bulk vertices.

### 3.2. Entanglement

In the following, different entanglement notions used in quantum information theory are recalled, in particular the von Neumann entropy and Rényi entropy.

The von Neumann entropy for a given normalized quantum state ρ is defined as

(4) $$S\left(\rho \right):=-\mathrm{Tr}\left(\rho \log\rho \right).$$

In physical systems with an exponential number of degrees of freedom, the computation is generally difficult. A generalization exists in which diagonalization of the density matrix ρ is not required. This definition, introduced by Rényi, is known as the Rényi entropy and defined as

(5) $$S_{n}\left(\rho \right):=\frac{1}{1-n}\log\left(\mathrm{Tr}\rho^{n}\right),$$

where it is well known that the Rényi entropy converges to the von Neumann entropy as n→1. The definitions provided above are for normalized quantum states; if the state is not normalized, one should first normalize it and then compute the entropy.

A subtlety regarding the upper bound on the rank of the reduced density matrix induced by the minimal cut should be mentioned here. A minimal cut is defined as the minimal number of edges in a graph that must be removed in order to fully separate a given fixed region of the graph. Although it is straightforward to observe that the rank of the reduced density matrix $\rho_{A}$ is upper-bounded by the local dimension *D* raised to the number of edges in the set *A*, i.e., $\mathrm{rank}(\rho_{A})\leq D^{\left|A\right|}$, a subtlety arises in that the rank of the reduced density matrix is upper-bounded by the minimum number of connecting edges, or the bottleneck (min-cut), rather than by the number of edges:

(6) $$\mathrm{rank}\left(\rho_{A}\right)\leq D^{F_{A}}$$

where $F_{A}$ is the min-cut (or the number of edges in the “bottleneck”); see, e.g., [8].

**Figure 2:**
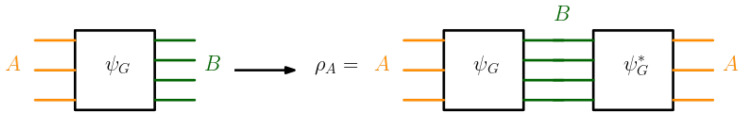
Constructing $\rho_{A}$ from $|\psi_{G}\rangle$ by tracing out the edges corresponding to the set *B*.

This can be demonstrated more clearly using an example. Consider a state $|\psi_{G}\rangle$ from which $\rho_{A}$ can be constructed, as shown in Figure 2.

**Figure 3:**
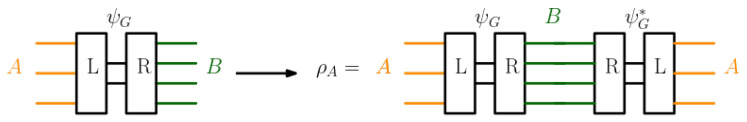
A factorization of the tensor $\psi_{G}$ yields a upper bound on the rank of the corresponding reduced density matrix $\rho_{A}$.

The internal structure of $|\psi_{G}\rangle$ can be considered by dividing the graph into two subgraphs, denoted as *L* and *R*, which are connected by the “bottleneck”, that is, the set of all edges for which their removal would disconnect the boundary sets *A* and *B*, see Figure 3.

Now, it is clear that $\mathrm{rank}(\rho_{A})\leq D^{F_{A}}$, where in this case $F_{A}=2$; consequently,

(7) $$S\left(\rho_{A}\right)\leq F_{A}\log D.$$

Having established the natural intuition around the role of the min-cut ($F_{A}$) in upper-bounding the entropy, the (maximal) flow approach for the random tensor network will be developed in the following sections.

## 4. Moment Computation

Given a random tensor network, we next investigate the behavior of entanglement between a specified subregion of the network and the remainder. To this end, we first address the moment computation of the quantum state ${\tilde{\rho }}_{A}$ for a subregion $A\subseteq E_{\partial }$. This initial computation will subsequently enable our analysis of the Rényi and von Neumann entropies in the following sections.

Let $A\subseteq E_{\partial }$ be a sub-boundary region of the graph *G*. The complementary region of *A* is denoted by $B:=E_{\partial }\setminus A$. The Hilbert spaces associated with the boundary regions *A* and *B* are denoted by $H_{A}:={⨂}_{e_{x}\in A}H_{e_{x}}$ and $H_{B}:={⨂}_{e_{x}\in B}H_{e_{x}}$, respectively.

In this work, we computed the *average entanglement entropy* at large bond dimensions:

(8) $${\tilde{\rho }}_{A}:=\frac{\rho_{A}}{\mathrm{Tr}\rho_{A}}\to \left\{\begin{array}{cc} \lim_{D\to \infty }E\;S_{n}\left({\tilde{\rho }}_{A}\right) & \\ \lim_{D\to \infty }E\;S\left({\tilde{\rho }}_{A}\right) \end{array}\right.$$

where ${\tilde{\rho }}_{A}$ is the normalized quantum state obtained by tracing out the region *B*, i.e., $\rho_{A}={\mathrm{Tr}}_{B}|\psi_{G}\rangle \langle \psi_{G}|$, with the partial trace over the Hilbert space $H_{B}$. In the above expression, the average is taken over all of the random Gaussian tensors at the vertices.

The first computation to be addressed here is the moment computation, as described in the following proposition. This computation will subsequently enable our calculation of the average entanglement entropy (Rényi and von Neumann entropies) in the limit D→∞, as analyzed in detail in the following sections. The result has previously been obtained in a very similar setting by Hastings [33] (Theorem 3, $E_{ind}$ ensemble); see also Section 5 of [8] for a closely related derivation.

**Proposition** **1.**  *For any $A\subseteq E_{\partial }$, it holds that*

(9) $$E\left[\mathrm{Tr}(\rho_{A}^{n})\right]=\sum_{\alpha =\left(\alpha_{x}\right)\in S_{n}^{\left|V\right|}}D^{n|E|-n|E_{b}|-H_{G}^{\left(n\right)}\left(\alpha \right)},\;\forall \;n\in N,$$

*where $H_{G}^{\left(n\right)}\left(\alpha \right)$ can be understood as the Hamiltonian of a classical “spin system” in which each spin variable takes a value from the permutation group $S_{n}$:*

(10) $$H_{G}^{\left(n\right)}\left(\alpha \right):=\sum_{(x,\cdot )\in A}|\gamma_{x}^{-1}\alpha_{x}|+\sum_{(x,\cdot )\in B}|{\mathrm{id}}_{x}^{-1}\alpha_{x}|+\sum_{\left(x,y\right)\in E_{b}}\left|\alpha_{x}^{-1}\alpha_{y}\right|.$$

Before providing the proof of the above proposition, we recall some properties of the permutation group $S_{n}$ and fixed the relevant notation. Here, the symbol $\gamma_{x}$ is used to denote the *total cycle* in the permutation group $S_{n}$ evaluated at (x,·)∈A:

$$\forall \left(x,\cdot \right)\in A,\;\gamma_{x}=\left(n\ldots 1\right).$$

Recall that a notion of distance in $S_{n}$, known as the *Cayley distance*, can be defined by

$$\begin{array}{cc} S_{n}\times S_{n} & \to R^{+}\\ d:(\alpha_{i},\alpha_{j}) & \to d\left(\alpha_{i},\alpha_{j}\right):=n-\#\left(\alpha_{i}^{-1}\alpha_{j}\right), \end{array}$$

where #(α) stands for the number of cycles in α. The distance $d(\alpha_{i},\alpha_{j})$ provides the minimum number of transpositions required to turn $\alpha_{i}$ into $\alpha_{j}$. In general, the distance in $S_{n}$ satisfies the triangle inequality, where

$$d\left(\alpha_{i},\alpha_{j}\right)\leq d\left(\alpha_{i},\sigma \right)+d\left(\sigma ,\alpha_{j}\right).$$

In particular, σ is said to be a *geodesic* between $\alpha_{i}$ and $\alpha_{j}$ in $S_{n}$ if $d\left(\alpha_{i},\alpha_{j}\right)=d\left(\alpha_{i},\sigma \right)+d\left(\sigma ,\alpha_{j}\right)$. The following notation for the distance is adopted instead of d(·,·), where

$$\left(\alpha_{i},\alpha_{j}\right)\in S_{n}\times S_{n},\;d\left(\alpha_{i},\alpha_{j}\right)=\left|\alpha_{i}^{-1}\alpha_{j}\right|.$$

**Proof.**  To prove the result announced in the proposition, it should first be remarked that we can use the well-known replica trick to write the trace on the left-hand side of Equation (9) as follows:

$$\mathrm{Tr}(\rho_{A}^{n})=\mathrm{Tr}\left(|\psi_{G}\rangle {\langle \psi_{G}|}^{⊗n}U_{\gamma_{A}}⊗{\mathrm{id}}_{B}\right).$$

The trace on the left-hand side (on $H_{A}$) may be rewritten as a full trace on *n* copies of the complete (bulk and boundary) Hilbert space, as shown on the right-hand side of the equation above. The notation $U_{\gamma_{A}}={⨂}_{(x,\cdot )\in A}U_{\gamma_{x}}$ is used to denote the tensor product of unitary representations of the permutation $\gamma_{x}=\left(n\ldots 1\right)\in S_{n}$ for each half-edge (x,·)∈A.

By expanding and taking the average over random Gaussian tensors, we obtain

$$\begin{array}{cc} E\mathrm{Tr}(\rho_{A}^{n}) & =\mathrm{Tr}\left(E\left[|\psi_{G}\rangle {\langle \psi_{G}|}^{⊗n}\right]\;U_{\gamma_{A}}⊗{\mathrm{id}}_{B}\right)\\ \; & =\mathrm{Tr}\;(\underset{e\in E_{b}}{⨂}|\Omega_{e}\rangle {\langle \Omega_{e}|}^{⊗n}E[\underset{x\in V}{⨂}|g_{x}\rangle {\langle g_{x}|}^{⊗n}]\;U_{\gamma_{A}}⊗{\mathrm{id}}_{B})\\ \; & =\mathrm{Tr}\;(\underset{e\in E_{b}}{⨂}|\Omega_{e}\rangle {\langle \Omega_{e}|}^{⊗n}\underset{x\in V}{⨂}E[|g_{x}\rangle {\langle g_{x}|}^{⊗n}]\;U_{\gamma_{A}}), \end{array}$$

where the shorthand notation $U_{\gamma_{A}}$ in the last equation above is used in place of $U_{\gamma_{A}}⊗{\mathrm{id}}_{B}.$ We recall the following property of random Gaussian states (see [34]):

$$\forall x\in V,\;E\left[|g_{x}\rangle {\langle g_{x}|}^{⊗n}\right]=\sum_{\left\{\alpha_{x}\right\}\in S_{n}}U_{\alpha_{x}}$$

where $U_{\alpha_{x}}$ is the unitary representation of $\alpha_{x}\in S_{n}$. Each permutation $\alpha_{x}\in S_{n}$ acts on each vertex in Hilbert space; hence, each acts implicitly on each half-edge incident to the vertex x∈V. Thus, the moment formula becomes

$$\begin{array}{cc} E\mathrm{Tr}(\rho_{A}^{n}) & =\sum_{\left\{\alpha_{x}\right\}\in S_{n}}\mathrm{Tr}\left[\underset{e\in E_{b}}{⨂}|\Omega_{e}\rangle {\langle \Omega_{e}|}^{⊗n}\underset{x\in V}{⨂}U_{\alpha_{x}}\;U_{\gamma_{A}}\right]\\ \; & =D^{-n|E_{b}|}\sum_{\left\{\alpha_{x}\right\}\in S_{n}}\prod_{(x,\cdot )\in A}D^{\#(\gamma_{x}^{-1}\alpha_{x})}\;\prod_{(x,\cdot )\in B}D^{\#({\mathrm{id}}_{x}^{-1}\alpha_{x})}\prod_{\left(x,y\right)\in E_{b}}D^{\#(\alpha_{x}^{-1}\alpha_{y})}, \end{array}$$

where the number of loops obtained by contracting the maximally entangled states (edges) upon taking the trace is counted by the formula above. The factor $D^{-n|E_{b}|}$ arises from the normalization associated with the contraction of the bulk edges (see Equation (2), where each edge contributes a factor of $D^{-1}$). By employing the relation between the Cayley distance and the number of loops, the result stated in the proposition is obtained. □

**Figure 4:**
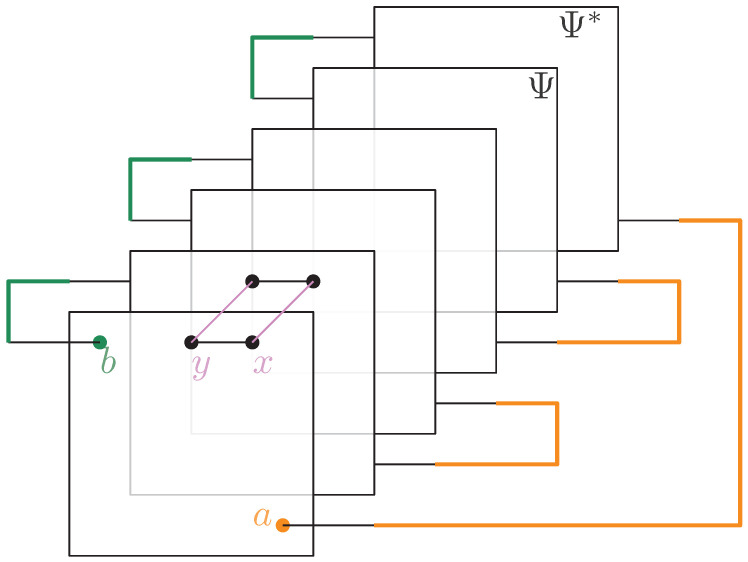
Graphical representation of the Wick theorem for the moment computation with n=3.

Graphically, the formula can be interpreted using Figure 4, where the case with n=3 is illustrated. By employing the graphical integration technique for Wick integrals as presented in [30,35], loops are obtained. Consequently, Cayley distances of three types arise: (a) between ${\mathrm{id}}_{x}$ and elements directly connected to it, originating from the region *B*; (b) between $\gamma_{x}$ and elements directly connected to it, originating from the region *A*; and (c) between elements neither directly connected to id nor γ, originating from the bulk. Based on this, the Hamiltonian can be rewritten in terms of Cayley distances, as follows:

(11) $$H_{G}^{\left(n\right)}\left(\alpha \right):=\sum_{(x,\cdot )\in A}|\gamma_{x}^{-1}\alpha_{x}|+\sum_{(x,\cdot )\in B}|{\mathrm{id}}_{x}^{-1}\alpha_{x}|+\sum_{\left(x,y\right)\in E_{b}}\left|\alpha_{x}^{-1}\alpha_{y}\right|$$

where (x,·)∈A represents the half-edges in *A*, (x,·)∈B, represents the half-edges in *B*, and $\left(x,y\right)\in E_{b}$ represents the edges in the bulk of the tensor network.

In the above proposition, only the numerator term of the normalized quantum state ${\tilde{\rho }}_{A}$ have been addressed. However, to compute the von Neumann and Rényi entropies (see Equations (4) and (5)), the state must be normalized and the moment computed.

The following proposition provides the moment computation of the normalization term in ${\tilde{\rho }}_{A}$.

**Proposition** **2.**  *For any $A\subseteq E_{\partial }$, it is found that*

(12) $$E\left[{\left(\mathrm{Tr}\rho_{A}\right)}^{n}\right]=\sum_{\alpha =\left(\alpha_{x}\right)\in S_{n}^{\left|V\right|}}D^{n|E|-n|E_{b}|-h_{G}^{\left(n\right)}\left(\alpha \right)},\;\forall \;n\in N,$$

*where the Hamiltonian $h_{G}^{\left(n\right)}\left(\alpha \right)$ is provided by*

(13) $$h_{G}^{\left(n\right)}\left(\alpha \right):=\sum_{\left(x,\cdot \right)\in E_{\partial }}|{\mathrm{id}}_{x}^{-1}\alpha_{x}|+\sum_{\left(x,y\right)\in E_{b}}\left|\alpha_{x}^{-1}\alpha_{y}\right|.$$

**Proof.**  The proof of this proposition follows directly from Proposition 1 by taking A=∅ such that $h_{G}^{\left(n\right)}\left(\alpha \right)$ is obtained as the special case of $H_{G}^{\left(n\right)}\left(\alpha \right)$ with A=∅. □

## 5. Asymptotic Behavior of Moments

In this section, the leading contributing terms of the moment as D→∞ are described using the *(maximal) flow* approach. First, the (maximal) flow approach is introduced, which allows for the estimation of the leading terms of the moments as D→∞; further details can be found in Proposition 5. Based on this result, the convergence of the moment as D→∞ to the moments of a graph-dependent measure $\mu_{G_{A|B}}$ can be deduced, as detailed in Theorem 4.

First, the results from the previous section are recalled. In Proposition 1, it was shown that the moments are provided by

$$E\mathrm{Tr}\left(\rho_{A}^{n}\right)=\sum_{\alpha =\left(\alpha_{x}\right)\in S_{n}^{\left|V\right|}}D^{n|E|-n|E_{b}|-H_{G}^{\left(n\right)}\left(\alpha \right)},$$

where the spin-valued Hamiltonian in the permutation group $S_{n}$ is provided by

$$H_{G}^{\left(n\right)}\left(\alpha \right):=\sum_{(x,\cdot )\in A}|\gamma_{x}^{-1}\alpha_{x}|+\sum_{(x,\cdot )\in B}|{\mathrm{id}}_{x}^{-1}\alpha_{x}|+\sum_{\left(x,y\right)\in E_{b}}\left|\alpha_{x}^{-1}\alpha_{y}\right|.$$

In particular, the contribution of the normalization term in ${\tilde{\rho }}_{A}$ (see Equation (8)) is the extended Hamiltonian $h_{G}^{\left(n\right)}\left(\alpha \right)$, as shown in Proposition 2 when one takes A=∅ in $H_{G}^{\left(n\right)}\left(\alpha \right)$.

The main goal of this section is to analyze the main contributing terms of the moment as D→∞. The leading terms will consist of solving the following minimization problem:

$$\min_{\alpha \in S_{n}^{\left|V\right|}}H_{G}^{\left(n\right)}\left(\alpha \right).$$

In particular, $h_{G}^{\left(n\right)}\left(\alpha \right)$ is minimized, yielding the leading contributing term as D→∞ for the normalization term of ${\tilde{\rho }}_{A}$. The minimization problem described above enables deduction of the moment convergence as D→∞ to the moment of the graph-dependent measure $\mu_{G_{A|B}}$ in Theorem 4.

The minimization problem above is addressed using the *(maximal)-flow* approach. This approach consists of first constructing a *network* $G_{A|B}$ from the original graph *G*. This network is constructed by first adding two extra vertices γ and id to *G* in such a way that all the half-edges associated with *A* are connected to the total cycle γ and that half-edges in *B* are connected to id. The network $G_{A|B}$ has the same bulk structure of *G*, with the difference that all the vertices in $G_{A|B}$ are valued in the permutation group $S_{n}$.

The flow approach is based on the identification of different augmenting paths in the network $G_{A|B}$, each starting from id and ending at γ. These various paths induce an *order* structure in $G_{A|B}$. By removing all augmenting paths in $G_{A|B}$, a lower bound for the extended Hamiltonian $H_{G_{A|B}}^{\left(n\right)}\left(\alpha \right)$ can be established; further details are provided in Proposition 3. Furthermore, it is demonstrated that the minimum is attained when the maximal flow from id to γ is achieved, as discussed in Proposition 5. In particular, it is shown that the minimum of the extended Hamiltonian $h_{G_{A|B}}^{\left(n\right)}\left(\alpha \right)$ is zero; see Proposition 6 for additional details.

Before the flow approach is introduced, it should be noted that the contributing terms of the moments at large dimensions were previously analyzed in [25] using the *(minimal) cut* approach. In that work, the existence of two *disjoint minimal cuts* in the graph (separating the region of interest from the remainder of the graph) was assumed to determine the contributions at large bond dimensions. In contrast, in the maximal flow approach presented here, no assumption regarding the existence of (minimal) cuts is made. By identifying various augmenting paths that achieve the maximal flow and applying the well-known maximal-flow minimal-cut theorem (see, e.g., [36], Theorem 8.6), the different minimal cuts can be deduced without any prior assumption.

**Definition** **4.**  *Let the network $G_{A|B}=\left(\tilde{V},\tilde{E}\right)$ be defined from the initial graph G=(V,E) such that*

$$\tilde{V}:=V⊔\left\{\mathrm{id},\gamma \right\}\;\mathrm{and}\;\tilde{E}:=E_{\tilde{A}}⊔E_{b}⊔E_{\tilde{B}},$$

*where the regions* $E_{\tilde{A}}$ *and* $E_{\tilde{B}}$ *is defined as follows:*

$$\begin{array}{cc} E_{\tilde{A}} & :=\underset{x\in V_{A}}{⨆}\left(x,\gamma \right)\\ E_{\tilde{B}} & :=\underset{x\in V_{B}}{⨆}\left(\mathrm{id},x\right) \end{array}$$

*where $V_{A}$ and $V_{B}$ respectively denote all of the vertices associated with the boundary region A and B. Moreover the vertices are valued in the permutation group $S_{n}$, where*

$$\forall x\in \tilde{V}\to \alpha_{x}\in S_{n}.$$

It should be remarked that in the above definition, the graph $G_{A|B}$ is constructed in such a way that all of the half-edges (x,·)∈A are connected to $\gamma =\left(n\cdots 1\right)\in S_{n}$ and that the half-edges (x,·)∈B are connected to id. Note also that there are no half-edges in $G_{A|B}$; the bulk region in the network $G_{A|B}$ remains the same as the one in graph *G*.

The extended Hamiltonian $H_{G_{A|B}}^{\left(n\right)}\left(\alpha \right)$ of $H_{G}^{\left(n\right)}\left(\alpha \right)$ in the network $G_{A|B}$ is provided by

(14) $$H_{G_{A|B}}^{\left(n\right)}\left(\alpha \right):=\sum_{x\in V_{A}}|\gamma^{-1}\alpha_{x}|+\sum_{x\in V_{B}}|{\mathrm{id}}^{-1}\alpha_{x}|+\sum_{\left(x,y\right)\in V_{b}}\left|\alpha_{x}^{-1}\alpha_{y}\right|,$$

where each term in the new Hamiltonian is valued in the network $G_{A|B}$. Moreover, the sums in the above formula are over the vertices $V_{A}$, $V_{B}$, and $V_{b}$, which are the vertices with the respective half-edges in the region *A*, *B*, and $E_{b}$.

As mentioned earlier, the flow approach consists of analyzing different paths that start from id and end in γ. This induces a natural orientation of the network $G_{A|B}$ (more precisely, a *poset structure*). In the following, the sets of different (edge-disjoint) paths in $G_{A|B}$ is defined.

**Definition** **5.**  *Let* $P(G_{A|B})$ *be the set of all possible paths from the source to the sink in* $G_{A|B}$*, where the source and sink are* id *and γ, respectively. Formally, the set of paths* $P(G_{A|B})$ *is defined as* 

$$P\left(G_{A|B}\right):=\left\{\pi_{i}:\;\pi_{i}:\;\mathrm{id}\to \gamma \right\},$$

*where* ${\left\{\pi_{i}\right\}}_{i}$ *are all the paths connecting* id *to* γ.

**Definition** **6.**  *Let* $\tilde{P}\left(G_{A|B}\right)$ *be the set of all families consisting of* edge-disjoint paths *in* $P(G_{A|B})$*:*

$$\tilde{P}\left(G_{A|B}\right):=\left\{\pi_{i}\in P\left(G_{A|B}\right):{\left\{\pi_{i}\right\}}_{i}\;\;\mathrm{are}\;\mathrm{edge}-\mathrm{disjoint}\;\right\}.$$

**Remark** **1.**  *It is clear from this definition that* $\tilde{P}\left(G_{A|B}\right)\subseteq P\left(G_{A|B}\right)$*.*

The process of searching for different paths that start from id and end at γ induces an ordering, more precisely a *poset structure*, in the network $G_{A|B}$. In the following, a definition of a poset structure is provided which will later be utilized in the maximal flow approach to minimize $H_{G_{A|B}}^{\left(n\right)}\left(\alpha \right)$.

**Definition** **7.**  *The poset structure* $P_{o}\left(G_{A|B}\right)$*is a* homogeneous *relation denoted by ≤ and satisfying the following conditions for all* $\alpha_{x},\alpha_{y},\alpha_{z}\in \tilde{V}$*:*

- *Reflexivity:* $\alpha_{x}\leq \alpha_{x}$*;*
- *Antisymmetry:* $\alpha_{x}\leq \alpha_{y}$ *and* $\alpha_{y}\leq \alpha_{x}$*, implying* $\alpha_{x}=\alpha_{y}$*;*
- *Transitivity: $\alpha_{x}\leq \alpha_{y}$ and $\alpha_{y}\leq \alpha_{z}$, implying $\alpha_{x}\leq \alpha_{z}$.*

**Definition** **8.**  *The* natural ordering *is defined as*

$$\mathrm{id}\leq \alpha_{1}\leq \alpha_{2}\leq \cdots \leq \alpha_{n}\leq \gamma$$

*for a path $\pi_{i}\in P\left(G_{A|B}\right)$ provided by*

$$\pi_{i}:\mathrm{id}\to \alpha_{1}\to \alpha_{2}\to \cdots \to \alpha_{n}\to \gamma .$$

Another useful notion in (maximal) flow analysis is the *permutation cluster*. A permutation cluster of a given permutation $\alpha_{x}$ is defined as all of the edge-connected permutations to $\alpha_{x}$.

**Definition** **9.**  *A* permutation cluster $\left[\alpha_{x}\right]$ *is defined as all the edge-connected permutations to* $\alpha_{x}\in S_{n}$*.*

**Remark** **2.**  *With the poset structure in* $G_{A|B}$*, a naturally-induced ordering is obtained in the cluster structures for each permutation connected to the permutation elements* ${\left\{\alpha_{i}\right\}}_{i\in \{x,y,z\}}$*, where all the properties of the above definition can be extended to the cluster* $\left[\alpha_{i}\right]$ *of a given permutation* $\alpha_{i}$*. More precisely, the following hold:*

$$\begin{array}{ccc} \alpha_{x}\leq \alpha_{y} & \Rightarrow \left[\alpha_{x}\right]\leq \left[\alpha_{y}\right];\\ \alpha_{x}=\alpha_{y} & \Rightarrow \left[\alpha_{x}\right]=\left[\alpha_{y}\right]; & \\ \alpha_{x}\leq \alpha_{y}\leq \alpha_{z} & \Rightarrow \left[\alpha_{x}\right]\leq \left[\alpha_{y}\right]\leq \left[\alpha_{z}\right]. \end{array}$$

**Definition** **10.**  *The max flow in $G_{A|B}$ is the maximum of all of the edge-disjoint paths in $\tilde{P}\left(G_{A|B}\right)$:*

$$\mathrm{maxflow}\left(G_{A|B}\right):=\max\left\{\left|\pi \right|\;:\;\pi \in \tilde{P}\left(G_{A|B}\right)\;\mathrm{is}\;a\;\mathrm{set}\;\mathrm{of}\;\mathrm{edge}-\mathrm{disjoint}\;\mathrm{paths}\right\}.$$

The following proposition provides a lower bound of the extended Hamiltonian $H_{G_{A|B}}\left(\alpha \right)$ that is saturated when the maximal flow in $G_{A|B}$ is achieved, as shown in Proposition 5. Similar ideas have been used by Hastings in Lemma 4 of [33] to lower-bound the moments of a random tensor network map.

**Proposition** **3.**  *Let* $\alpha \in S_{n}^{\left|V\right|}$ *and* $\tilde{P}\left(G_{A|B}\right)$ *be an arbitrary set of edge-disjoint paths in* $G_{A|B}$ *and set* $k:=|\tilde{P}\left(G_{A|B}\right)|$*; then, the following inequality holds:*

(15) $$H_{G_{A|B}}^{\left(n\right)}\left(\alpha \right)\geq k\left(n-1\right)+H_{G_{A|B}\setminus {⨆}_{i\in [k]}\pi_{i}}^{\left(n\right)}\left(\alpha \right)\geq k\left(n-1\right)$$

*where* $H_{G_{A|B}\setminus {⨆}_{i\in [k]}\pi_{i}}^{\left(n\right)}\left(\alpha \right)$ *, defined as follows:*

(16) $$H_{G_{A|B}\setminus {⨆}_{i\in [k]}\pi_{i}}^{\left(n\right)}\left(\alpha \right):=\sum_{x\in V_{A}\setminus {⨆}_{i\in [k]}\pi_{i}}|\gamma^{-1}\alpha_{x}|+\sum_{x\in V_{B}\setminus {⨆}_{i\in [k]}\pi_{i}}|{\mathrm{id}}^{-1}\alpha_{x}|+\sum_{x∼y\in V_{b}\setminus {⨆}_{i\in [k]}\pi_{i}}\left|\alpha_{y}^{-1}\alpha_{x}\right|.$$

It should be mentioned that the sums in the above proposition are over $\beta \setminus {⨆}_{i\in [k]}\pi_{i}$ for $\beta \in \{V_{A},V_{B},V_{b}\}$, which make up the set of the different boundary and bulk regions when removing all the different edges and vertices that will contribute in different paths $\pi_{i}\in \tilde{P}\left(G_{A|B}\right)$ in $G_{A|B}$.

**Proof.**  Consider a set of edge disjoint paths ${\left\{\pi_{i}\right\}}_{i\in [k]}\in \tilde{P}\left(G_{A|B}\right)$. We can fix a path $\pi_{i}$ for a given i∈[k] where

$$\pi_{i}:\mathrm{id}\to \alpha_{x_{1}}\to \alpha_{x_{2}}\to \cdots \to \alpha_{x_{n}}\to \gamma$$

is a path that starts from id, explores ${\left\{x_{i}\right\}}_{i\in [n]}$ vertices, and ends in γ. Using Equation (14) with the path defined above, we obtain

$$H_{G_{A|B}}^{\left(n\right)}\left(\alpha \right)=|\alpha_{x_{1}}|+\sum_{i=1}^{n-1}|\alpha_{x_{i}}^{-1}\alpha_{x_{i+1}}\left|+\right|\alpha_{x_{n}}^{-1}\gamma |+H_{G_{A|B}\setminus \pi_{i}}^{\left(n\right)}\left(\alpha \right)\geq n-1+H_{G_{A|B}\setminus \pi_{i}}^{\left(n\right)}\left(\alpha \right),$$

where we have used the triangle inequality for the Cayley distance together with the fact that |γ|=n−1. The Hamiltonian $H_{G_{A|B}\setminus \pi_{i}}^{\left(n\right)}\left(\alpha \right)$ is the contribution when the path $\pi_{i}$ from $G_{A|B}$ is used.

By iteration over all the edge-disjoint paths ${\left\{\pi_{i}\right\}}_{i\in [k]}\in \tilde{P}\left(G_{A|B}\right)$, the desired result is obtained. The second inequality is obtained by observing that $H_{G_{A|B}\setminus \pi_{i}}^{\left(n\right)}\left(\alpha \right)\geq 0$, which ends the proof of this proposition. □

**Proposition** **4.**  *Given a graph G, there exist a tuple of permutations α such that $H_{G_{A|B}}^{\left(n\right)}\left(\alpha \right)=\mathrm{maxflow}\left(G_{A|B}\right)$.*

**Proof.**  By the celebrated max-flow min-cut theorem, the maximum flow in the network is equal to its minimal cut. Recall that a *cut* of a network is a partition of its set of vertices into two subsets S∋s and T∋t, with the size of the cut being the number of S−T edges. In the current setting, the max-flow min-cut theorem (see, e.g., [36], Theorem 8.6) implies that there exists a partition of the vertex set $\tilde{V}$ of $G_{A|B}$ (see Definition 4) into two subsets $\tilde{V}=S⊔T$ with id∈S and γ∈T, such that

$$\mathrm{maxflow}\left(G_{A|B}\right)=|\left\{\left(x,y\right)\in \tilde{E}\;:\;x\in S\;\mathrm{and}\;y\in T\right\}|.$$

For x∈V, we define

$$\alpha_{x}=\left\{\begin{array}{cc} \mathrm{id} & \;\;\mathrm{if}\;x\in S\\ \gamma & \;\;\mathrm{if}\;x\in T. \end{array}\right.$$

**Figure 5:**
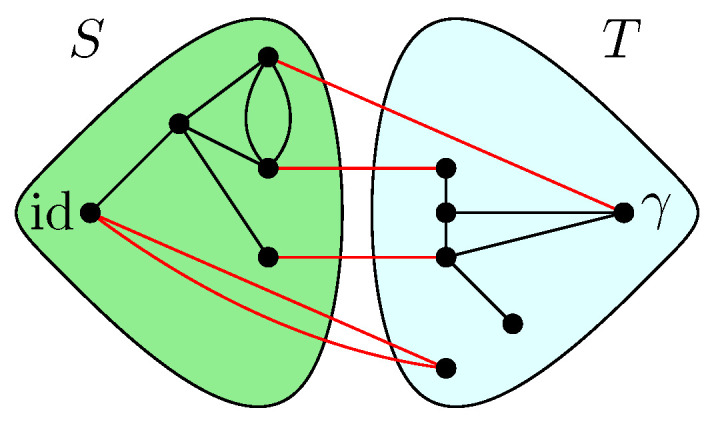
The different edges corresponding to a cut S−T in the network $G_{A|B}$.

Because id∈S and γ∈T, we have

$$\begin{array}{cc} H_{G_{A|B}}^{\left(n\right)}\left(\alpha \right) & =\sum_{x\in V_{A}}|\gamma^{-1}\alpha_{x}|+\sum_{x\in V_{B}}|{\mathrm{id}}^{-1}\alpha_{x}|+\sum_{\left(x,y\right)\in V_{b}}\left|\alpha_{x}^{-1}\alpha_{y}\right|\\ \; & =\sum_{\begin{array}{c} x\in V_{A}\\ x\in S \end{array}}|\gamma^{-1}\alpha_{x}|+\sum_{\begin{array}{c} x\in V_{B}\\ x\in T \end{array}}|{\mathrm{id}}^{-1}\alpha_{x}|+\sum_{\begin{array}{c} \left(x,y\right)\in V_{b}\\ x\in S\;\mathrm{and}\;y\in T \end{array}}\left|\alpha_{x}^{-1}\alpha_{y}\right|\\ \; & =(n-1)[|\{(x,\cdot )\in A\;:\;x\in S\}|+|\{(x,\cdot )\in B\;:\;x\in T\}|+\\ \; & \;\;\;|\{\left(x,y\right)\in E_{b}\;:\;x\in S\;\mathrm{and}\;y\in T\}|]\\ \; & =\left(n-1\right)\mathrm{maxflow}\left(G_{A|B}\right), \end{array}$$

where in the last claim we have used the fact that there are no edges between id and γ in $\tilde{E}$; see Figure 5. □

**Proposition** **5.**  *For all n≥1, it holds that*

$$\min_{\alpha \in S_{n}^{\left|V\right|}}H_{G_{A|B}}^{\left(n\right)}\left(\alpha \right)=\left(n-1\right)\mathrm{maxflow}\left(G_{A|B}\right).$$

**Proof.**  This proof follows from the two previous propositions. □

When all the augmenting paths in the network $G_{A|B}$ achieving the maximal flow have been identified and removed, a *clustered graph* $G_{A|B}^{c}$ is obtained by identifying different remaining connected permutations.

The following example provides an illustration of the different steps described above to analyze the maximum flow problem in the case of the tensor network represented in Figure 1.

**Example** **2.**  *Figure 7 represents the network* $G_{A|B}$ *associated with the random tensor network from Figure 1. The vertices in the network are valued in the permutation group* $S_{n}$*. The network is constructed by adding two extra vertices γ and* id *by connecting all of the half-edges in A to γ and the half-edges in B to* id*. The flow approach induces a flow from* id *to γ; here, the maximum flow in Figure 7 is 4, where the augmenting paths achieving it are shown in color. By removing the four edge-disjoint augmenting paths, we obtain the clustered graph* $G_{A|B}^{c}$ *in Figure 8 by identifying the remaining connected edges as a single permutation cluster (i.e.,* id *with* $\alpha_{15}$*)* *to form the cluster* [id,15]*.*

**Theorem** **4.**  *In the limit* D→∞*, for all* n≥1 *we have the following:*

$$\lim_{D\to \infty }E\frac{1}{D^{F(G_{A|B})}}[\mathrm{Tr}\left({\left(D^{F\left(G_{A|B}\right)-\left|E_{\partial }\right|}\;\rho_{A}\right)}^{n}\right)]=m_{n}$$

*where* $m_{n}$ *is the number of permutations achieving the minimum of the network Hamiltonian* $G_{A|B}^{\left(n\right)}$*. These numbers are the moments of a probability measure* $\mu_{G_{A|B}}$ *which is compactly supported on* [0,+∞)*.*

**Proof.**  For fixed *n*, the convergence to $m_{n}$ (the number of minimizers of the Hamiltonian $G_{A|B}^{\left(n\right)}$) follows from Proposition 1 and Proposition 5. The claim that the numbers ${\left(m_{n}\right)}_{n}$ are the moments of a compactly supported probability measure follows basically from Prokhorov’s theorem ([37], Section 5; see also [33], Footnote 2). Indeed, we note that at fixed *D*, the quantity

$$E\frac{1}{D^{F(G_{A|B})}}\left[\mathrm{Tr}\left({\left(D^{F\left(G_{A|B}\right)-\left|E_{\partial }\right|}\;\rho_{A}\right)}^{n}\right)\right]$$

is the *n*-th moment of the empirical eigenvalue distribution of the random matrix $D^{F\left(G_{A|B}\right)-\left|E_{\partial }\right|}\;\rho_{A}$, restricted to a subspace of dimension $D^{F(G_{A|B})}$ containing its support (this follows from the fact that $D^{F(G_{A|B})}$ is an upper bound on the rank of $\rho_{A}$; see Equation (6)). These measures have finite second moments, meaning that the sequence (index by *D*) is tight. The limiting moments satisfy Carleman’s condition, since $m_{n}\leq {\mathrm{Cat}}_{n}^{\left|V\right|}$, proving that the limit measure $\mu_{G_{A|B}}$ has compact support; recall that ${\mathrm{Cat}}_{n}\leq 4^{n}$ is the *n*-th *Catalan number* (see Appendix A). Because the matrix $\rho_{A}$ is positive semi-definite, $\mu_{G_{A|B}}$ must be supported on [0,+∞). □

**Remark** **3.**  *The obtained moments are provided by a graph-dependent measure. It will be shown in the following sections that such measures can be explicitly constructed if the partial order* $G_{A|B}^{o}$ *is series-parallel; see Section 6 and Theorem 5 for more details.*

In the above, the contribution terms at a large bond dimension D→∞ of $E\mathrm{Tr}(\rho_{A}^{n})$ are those that minimize $H_{G_{A|B}}^{\left(n\right)}\left(\alpha \right)$. As shown in Proposition 5, the quantity $H_{G_{A|B}}^{\left(n\right)}\left(\alpha \right)$ is minimized when the maximal flow is attained in $G_{A|B}$.

For later purposes, if the moment of ${\tilde{\rho }}_{A}$ is to be analyzed, then the contribution of the normalization term of ${\tilde{\rho }}_{A}$ at large bond dimension should also be considered. Recall from Proposition 2 that the contribution of the normalization term is provided by

$$E\left[{\left(\mathrm{Tr}\rho_{A}\right)}^{n}\right]=\sum_{\alpha =\left(\alpha_{x}\right)\in S_{n}^{\left|V\right|}}D^{n|E|-n|E_{b}|-h_{G}^{\left(n\right)}\left(\alpha \right)},\;\forall \;n\in N,$$

where

$$h_{G}^{\left(n\right)}\left(\alpha \right):=\sum_{\left(x,\cdot \right)\in E_{\partial }}|{\mathrm{id}}_{x}^{-1}\alpha_{x}|+\sum_{\left(x,y\right)\in E_{b}}\left|\alpha_{x}^{-1}\alpha_{y}\right|.$$

At a large dimension D→∞, the contributed terms are provided by the one that will minimize the extended Hamiltonian $h_{G_{A|B}}^{\left(n\right)}\left(\alpha \right)$ in $G_{A|B}$:

$$h_{G_{A|B}}^{\left(n\right)}\left(\alpha \right):=\sum_{x\in V_{\partial }}|{\mathrm{id}}^{-1}\alpha_{x}|+\sum_{\left(x,y\right)\in V_{b}}\left|\alpha_{x}^{-1}\alpha_{y}\right|,$$

where the first is over all the vertices $V_{\partial }$ with boundary edges and where $V_{b}$ are the bulk vertices.

**Proposition** **6.**  *Let $h_{G_{A|B}}^{\left(n\right)}\left(\alpha \right)$ be the extended Hamiltonian in $G_{A|B}$; then, for all n≥1 we have*

$$\min_{\alpha \in S_{n}^{|\tilde{V}|}}h_{G_{A|B}}^{\left(n\right)}\left(\alpha \right)=0,$$

*which is achieved by identifying all the permutations with* id*.*

**Proof.**  To minimize the Hamiltonian $h_{G_{A|B}}^{\left(n\right)}\left(\alpha \right)$, the same approach is followed; all half-edges *A* are connected to γ and all half-edges *B* to id. However, in $h_{G_{A|B}}^{\left(n\right)}\left(\alpha \right)$ all of the boundary terms are connected to id, meaning that no path starts from id and ends in γ. Per the bulk connectivity of *G*, the minimum is achieved by identifying all of the permutations with id; thus, the result follows from Proposition 5. □

**Remark** **4.**  *In Proposition 6, the Hamiltonian* $h_{G_{A|B}}^{\left(n\right)}\left(\alpha \right)$ *is obtained by taking* A=∅ in $H_{G}^{\left(n\right)}\left(\alpha \right)$ *(see Equation* (10)*). It should be mentioned that if* $B=E_{\partial }\setminus A=\emptyset$*, then the same form of the Hamiltonian* $h_{G_{A|B}}^{\left(n\right)}\left(\alpha \right)$ *is obtained, where instead of all the half-edges being connected to* id*, they are all connected to γ. Therefore, it can be deduced that there are no paths that start from id and end at γ; hence, the minimum is 0, which is achieved by identifying all the permutations with γ.*

**Corollary** **1.**  *For any* $A\subseteq E_{\partial }$ *moments of the normalization term, it converges to* 1*; more precisely,*

$$\lim_{D\to \infty }E{\left(\mathrm{Tr}\left(D^{-|E_{\partial }|}\rho_{A}\right)\right)}^{n}=1.$$

**Proof.**  By taking the average, as shown in Proposition 2, we obtain the Hamiltonian $h_{G}^{\left(n\right)}\left(\alpha \right)$. Per the maximal flow, the Hamiltonian is minimized by identifying all of the permutations to id; therefore, we have $F(G_{A|B})=0$, as shown in Proposition 6. By removing all of the augmenting paths that achieve maximal flow, the obtained residual graph is trivial, with only two disjoint vertices γ and the identity cluster [id]. Hence, by Theorem 4 we obtain the desired result. □

## 6. Moment for Ordered Series-Parallel Network

In this section, the notion of a series-parallel graph (Section 6.1) is introduced. This notion allows the moment to be computed as an explicit graph-dependent measure (Section 6.2). More precisely, it is show that with the help of free probability, the corresponding graph-dependent measure can be explicitly constructed in the case where the obtained partial order $G_{A|B}^{o}$ is series-parallel.

### 6.1. Series-Parallel Graph

In this subsection, the notion of series-parallel partial orders is introduced, which in the following subsection will allow for explicit computation of the moments as graph-dependent measures.

First, the notion of series-parallel partial order [38] is recalled and some crucial definitions are provided that will play an important role in the rest of this section.

Given two partial orders $\left(P_{i},\leq_{i}\right)$, i=1,2, we defines their *series* (resp. *parallel*) composition as follows: the base set is $P:=P_{1}⊔P_{2}$ and the order relation x≤y if

- $x,y\in P_{i}$ and $x\leq_{i}y$ *or* $x\in P_{1}$ and $y\in P_{2}$ in the series case;
- $x,y\in P_{i}$ and $x\leq_{i}y$ in the parallel case.

It is more convenient to represent partial orders by their *covering graphs*, where an oriented graph G(V,E) is associated with a partial order (P,≤), with V=P and x→y∈E if and only if x<y and there does not exist *z* such that x<z<y. Recall that x<y denotes x≤y and x≠y. The series and parallel composition for partial orders can be elegantly interpreted in terms of directed graphs (or networks, in this context). In what follows, the terms “partial order” and “partial order graph” are used interchangeably.

**Figure 6:**
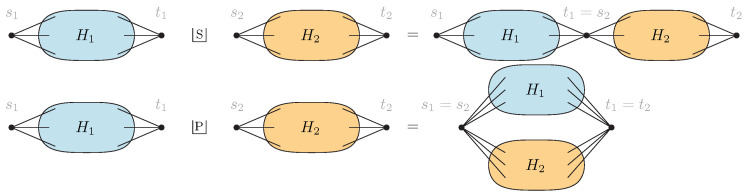
Series (**top**) and parallel (**bottom**) composition of two networks (graphs) $H_{1,2}$.

**Definition** **11**  ([38])**.** *Let* $H_{1}$ *and* $H_{2}$ *be two directed graphs with their respective sources* $s_{i}$ *and sinks* $t_{i}$ *for* i∈{1,2}*. A* series-parallel network *is a directed graph* G=(V,E) *containing two distinct vertices* s≠t∈V*, called the* source *and the* sink*, that can be obtained recursively from the trivial network* $G_{\mathrm{triv}}=\left(\left\{s,t\right\},\left\{\left\{s,t\right\}\right\}\right)$ *using the following two operations (see Figure 6):*

- *Series concatenation: G=H1SH2 is obtained by identifying the sink of $H_{1}$ with the source of $H_{2}$, i.e., $t_{1}=s_{2}$.*
- *Parallel concatenation: G=H1PH2 is obtained by identifying the source and the sink of $H_{1}$ and $H_{2}$, i.e., $s_{1}=s_{2}$ and $t_{1}=t_{2}$.*

**Remark** **5.**  *Note that the parallel concatenation is a commutative operation, while the series concatenation is in general not commutative:*

∀G,HGPH=HPG,ingeneralGSH≠HSG.

From a given series-parallel network, different probability distributions can be associated that are constructed from the parallel and series concatenation introduced in Definition 11.

**Definition** **12.**  *A probability measure $\mu_{G}$ is associated with a series-parallel network G, defined recursively as follows:*

- *The Dirac mass at* 1 *is associated with the trivial network $G_{\mathrm{triv}}=\left(\left\{s,t\right\},\left\{\left\{s,t\right\}\right\}\right)$:*
  $$\mu_{G_{\mathrm{triv}}}:=\delta_{1}.$$
- *For series concatenation, the free multiplicative convolution of the parts is taken along with the measure MP:*
μGSH:=μG⊠MP⊠μH.
- *For parallel concatenation, the classical multiplicative convolution of the parts is taken:*
μGPH:=μG×μH.

In the above definition, the free product convolution ⊠ and the Marc̆henko–Pastur distribution MP are used; refer to Appendix A for a self-contained introduction to free probability theory.

### 6.2. Moment as Graph-Dependent Measure

In this subsection, with the help of the series-parallel notion introduced in the previous subsection, we show that the moments $m_{n}$ in Theorem 4 are explicitly constructed from a graph-dependent measure in the case where the obtained partial order $G_{A|B}^{o}$ is series-parallel.

Before presenting the results of this subsection, the different results obtained in the previous sections are first recalled. For a given random tensor network, as represented for example in Figure 1, the moment for a normalized quantum state corresponding to a given subregion $A\subseteq E_{\partial }$ of the graph was computed in Section 4 (see Propositions 1 and 2). The evaluation of the moment as D→∞ was approached via the maximal flow method, as analyzed in Section 5. The network $G_{A|B}$ was constructed from the graph *G* by connecting each of the regions *A* and *B* to γ and id, respectively, where the flow consists of analyzing the different paths starting from id and ending in γ. By removing all of the different augmenting paths achieving maximal flow, a clustered graph $G_{A|B}^{c}$ remains that is obtained by identifying different edge-connected permutations, as represented in Figure 8 for the clustered graph associated with the network $G_{A|B}$ in Figure 1. Using the maximal flow, Proposition 5 allows us to show the convergence of the moments provided by a graph-dependent measure $\mu_{G_{A|B}}$, as stated in Theorem 4. Moreover, from Proposition 6 it is deduced in Corollary 1 that the normalization terms converge to 1.

From the clustered graph $G_{A|B}^{c}$, a *partial order* $G_{A|B}^{o}$ is constructed in which the vertices in $G_{A|B}^{o}$ correspond to the different permutation clusters. Figure 9 illustrates the obtained partial order $G_{A|B}^{o}$ for the network $G_{A|B}$ shown in Figure 1. If the partial order $G_{A|B}^{o}$ is series-parallel (see Definition 11), convergence in moments of ${\tilde{\rho }}_{A}$ to an explicit partial order measure $\mu_{G_{A|B}^{o}}$ occurs, which will be explicitly shown in the following subsections.

The following theorem shows the convergence to a moment-dependent measure $\mu_{G_{A|B}^{o}}$ in the case where the obtained partial order $G_{A|B}^{o}$ is series-parallel.

**Theorem** **5.**  *For any $A\subseteq E_{\partial }$, assuming that the partial order $G_{A|B}^{o}$ is series-parallel, the limit measure from Theorem 4 can be explicitly constructed from the partial order*

$$\mu_{G_{A|B}}=\mu_{G_{A|B}^{o}}.$$

*In particular, the moments of the reduced tensor network matrix are provided by*

(17) $$\lim_{D\to \infty }\frac{1}{D^{F(G_{A|B})}}E\mathrm{Tr}\left({\left(D^{F\left(G_{A|B}\right)-\left|E_{\partial }\right|}\rho_{A}\right)}^{n}\right)=\int t^{n}\;d\mu_{G_{A|B}^{o}}.$$

**Proof.**  It suffices to show that the numbers $m_{n,G_{A|B}^{o}}$ are the moments of the probability measure $\mu_{G_{A|B}^{o}}$. This will be proved using the recursive structure of the series-parallel networks (see Definition 11) together with that of the probability measure $\mu_{G_{A|B}^{o}}$ (see Definition 12).

If the partial order $G_{A|B}^{o}$ is trivial, then it consists only of two connected components, namely, that of the identity (source) [id] and the sink [γ]. Hence, all the permutations associated with the connected components are fixed to be either id or γ. Thus, $m_{n,G_{A|B}^{o}}=1$ for all n≥1, which are the moments of the measure $\mu_{G_{A|B}^{o}}=\delta_{1}$. In this way, the claim is seen to hold for the initial case of a trivial network.

If the partial order $G_{A|B}^{o}$ is the *parallel* concatenation of two networks GA|Bo=H1PH2 having the same source and sink as $G_{A|B}^{o}$, then the geodesic equalities for $G_{A|B}^{o}$ are the disjoint union of the geodesic equalities for the vertices in $H_{1}$ and those for the vertices of $H_{2}$. In turn, this implies that

$$m_{n,G_{A|B}^{o}}=m_{n,H_{1}}\cdot m_{n,H_{2}}$$

for all n≥1, as there is no geodesic inequality mixing vertices from $H_{1}$ with vertices in $H_{2}$. Hence, by the induction hypothesis, we have

mn,GA|Bo=∫tndμH1·∫tndμH2=∫tndμH1PμH2=∫tndμGA|Bo,

proving the claim for the parallel concatenation of networks.

Finally, the case where the network is the *series* concatenation of two networks GA|Bo=H1SH2 is considered. This means that there is a connected component, say [β], which is common to the two networks, as it is the sink of $H_{1}$ and the source of $H_{2}$. All geodesic equality conditions for $H_{1}$ are of the form

$$\mathrm{id}\to \alpha_{1}^{\left(1\right)}\to \cdots \to \alpha_{k_{1}}^{\left(1\right)}\to \beta ,$$

while those of $H_{2}$ are of the form

$$\beta \to \alpha_{1}^{\left(2\right)}\to \cdots \to \alpha_{k_{2}}^{\left(2\right)}\to \gamma .$$

In particular, the geodesic equality conditions for GA|Bo=H1SH2 are of the form

$$\mathrm{id}\to \alpha_{1}^{\left(1\right)}\to \cdots \to \alpha_{k_{1}}^{\left(1\right)}\to \beta \to \alpha_{1}^{\left(2\right)}\to \cdots \to \alpha_{k_{2}}^{\left(2\right)}\to \gamma .$$

The variable β is a non-constrained non-crossing partition of [n], and summing over it corresponds to taking the free multiplicative convolution with respect to the Marc̆henko–Pastur distribution:

mn,GA|Bo=∑β∈NC(n)αi(1)≤β≤αj(2)1=∫tndμH1⊠MP⊠μH2=∫tndμH1SH2=∫tndμGA|Bo.

This proves the final claim and concludes the proof. □

**Figure 7:**
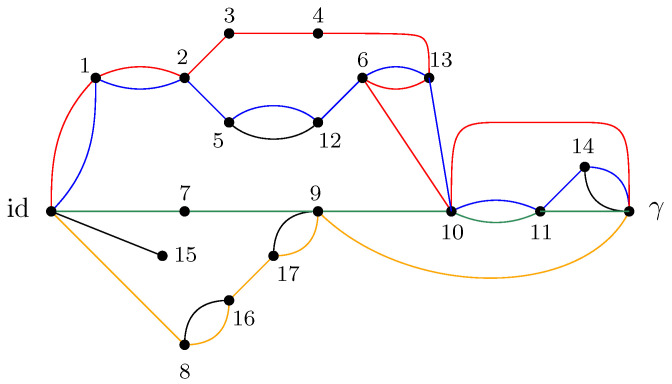
The network associated with the random tensor network marginal from Figure 1. The maximum flow of this network (with source id and sink γ) is 4. The four augmenting paths achieving this value are shown in color.

**Figure 8:**
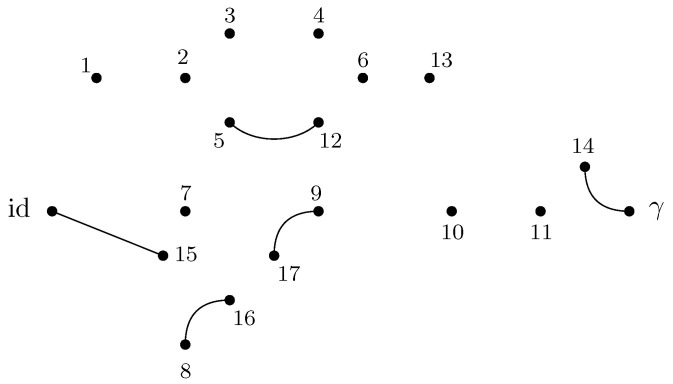
The clustered network corresponding to Figure 7 obtained by removing the four edge-disjoint augmenting paths.

**Figure 9:**
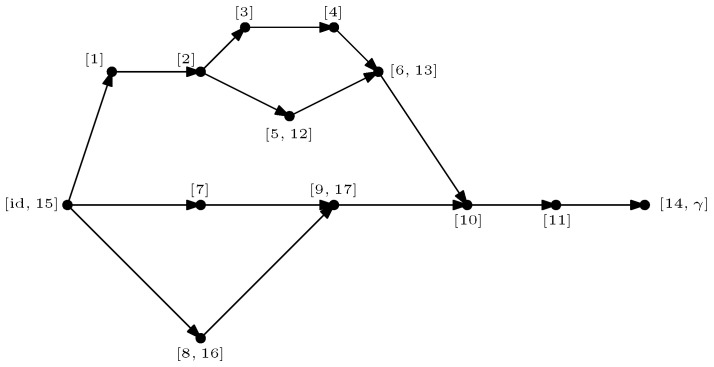
The order graph corresponding to the networks from Figure 1. The partial order relations are to be read from left to right. The elements of this partial-order relation are the connected components of the clustered network from Figure 8, which are eventually identified after taking into account the inequalities from the augmenting paths from Figure 7.

**Example** **3.**  *As an example, the graph illustrated in Figure 1 is considered. As described in the previous sections, the dominant terms of moments in Proposition 1 are obtained by analyzing the maximum flow in $G_{A|B}$, as shown in Figure 7, where $\mathrm{maxflow}(G_{A|B})=4$. The partial order $G_{A|B}^{o}$ obtained by removing from $G_{A|B}$ those edges that participate in the maximum flow, is depicted in Figure 8. Using the four augmenting paths (displayed by the colors in Figure 7), the* partial order *on the connected components of the partial order is constructed, as depicted in Figure 9.*

*This process is fundamental to the presented approach; the details for one of these geodesics are provided next. For example, consider the augmenting path*

id→1→2→3→4→13→6→10→γ

*depicted in red in Figure 7. Because the respective pair of points (id,15) and 14,γ in the clustered graph from Figure 8 are in the same connected components (clusters), this augmenting path gives rise to the following list of partial order relations:*

[id,15]⪯[1]⪯[2]⪯[3]⪯[4]⪯[13]⪯[6]⪯[10]⪯[14,γ].

*The other three augmenting paths, respectively depicted in blue, green, and orange in Figure 7, give rise to the following list of inequalities:*

$$\begin{array}{cc} \left[\mathrm{id},15\right] & ⪯[1]⪯[2]⪯[5,12]⪯[6]⪯[13]⪯[10]⪯[11]⪯[14,\gamma ]\\ \left[\mathrm{id},15\right] & ⪯[7]⪯[9,17]⪯[10]⪯[11]⪯[14,\gamma ]\\ \left[\mathrm{id},15\right] & ⪯[8,16]⪯[9,17]⪯[14,\gamma ]. \end{array}$$

*The partial order depicted in Figure 9 is compiled from the set of inequalities coming from the (fixed) list of augmenting paths yielding the maximum flow (here, 4). It is important to note that some connected components (clusters) can be identified in this partial order due to the* anti-symmetry *property x⪯yandy⪯x⇒x=y; in our example, this happens for the clusters [6] and [13].*

*As an application of Theorem 5, it is possible to provide explicit moments of the measure $\mu_{G_{A|B}^{o},A}$:*

(18) $$\lim_{D\to \infty }ED^{-4}\mathrm{Tr}\left[{\left(D^{-6}\rho_{A}\right)}^{n}\right]=m_{n,G_{A|B}^{o}}=\int x^{n}\;d\mu_{G_{A|B}^{o}}.$$

*The powers of D in the normalization follow from $|E_{\partial }|=10$ (see the boundary edges in Figure 1) and from $\mathrm{maxflow}(G_{A|B})=4$. The resulting probability measure $\mu_{G_{A|B}^{o}}$ associated with the partial order from Figure 9 is provided by*

(19) $$\mu_{G_{A|B}^{o}}=\left\{\left[{\mathrm{MP}}^{⊠3}⊠\left({\mathrm{MP}}^{⊠2}\times \mathrm{MP}\right)\right]\times \left[(\mathrm{MP}\times \mathrm{MP})⊠\mathrm{MP}\right]\right\}⊠{\mathrm{MP}}^{⊠2}.$$

**Figure 10:**
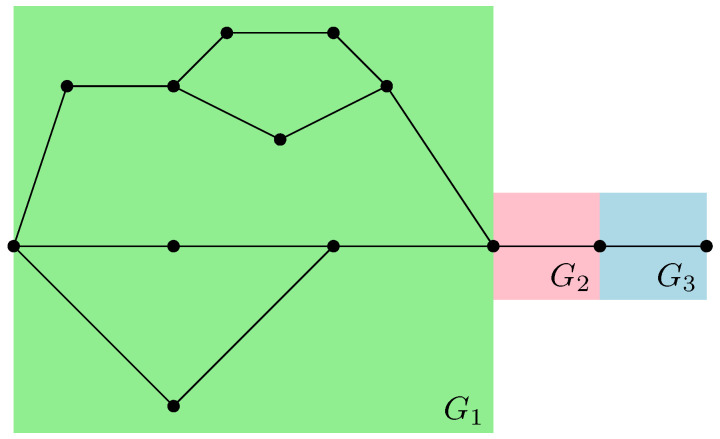
Representation of the partial order $G_{A|B}^{o}$ factorizes to a series combination of graphs $G_{1}$, $G_{2}$, and $G_{3}$.

*The above measure is obtained by the iterative procedure from Definition 12 as follows. First, observe that the graph in Figure 9 can be decomposed as a* series *composition of three graphs G1SG2SG3; hence, using $\mu_{G_{2}}=\mu_{G_{3}}=\delta_{1}$, it follows that (see Figure 10)*

$$\mu_{G_{A|B}^{o}}=\mu_{G_{1}}⊠\mathrm{MP}⊠\mu_{G_{2}}⊠\mathrm{MP}⊠\mu_{G_{3}}=\mu_{G_{1}}⊠{\mathrm{MP}}^{⊠2}.$$

**Figure 11:**
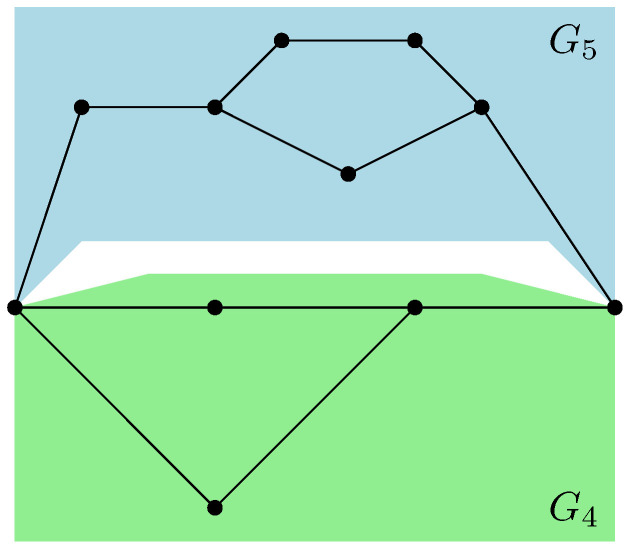
The graph $G_{1}$ is factorized to the parallel composition of $G_{4}$ and $G_{5}$.

*Now, we can observe that $G_{1}$ is a* parallel *composition of two other graphs; hence (see Figure 11)*

$$\mu_{G_{1}}=\mu_{G_{4}}\times \mu_{G_{5}}.$$

**Figure 12:**
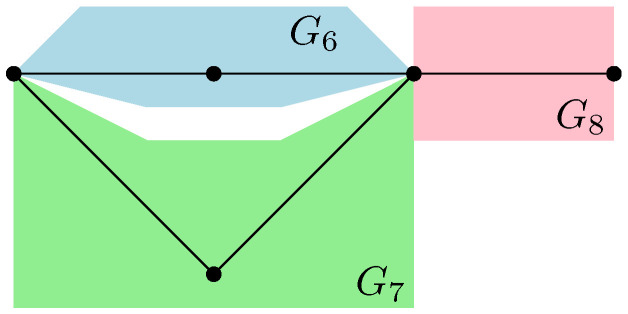
The graph $G_{4}$ is factorized to the series composition of the graph $G_{8}$, with graph $G_{6}$ parallel to $G_{7}$.

*The graphs $G_{4}$ and $G_{5}$ are now analyzed separately. First, $G_{4}$ can be decomposed as a series composition between the parallel composition of $G_{6}$ and $G_{7}$, and $G_{8}$, that is (see Figure 12)*

G4=G6PG7SG8⇒μG4=μG6×μG7⊠MP⊠μG8.

*Now,* $G_{6}$ *and* $G_{7}$ *are series compositions of two trivial graphs; thus,* $\mu_{G_{6}}=\mu_{G_{7}}=\mathrm{MP}$ *and* $\mu_{G_{8}}=\delta_{1}$*. It follows that*

$$\mu_{G_{4}}=\left(\mathrm{MP}\times \mathrm{MP}\right)⊠\mathrm{MP}.$$

**Figure 13:**
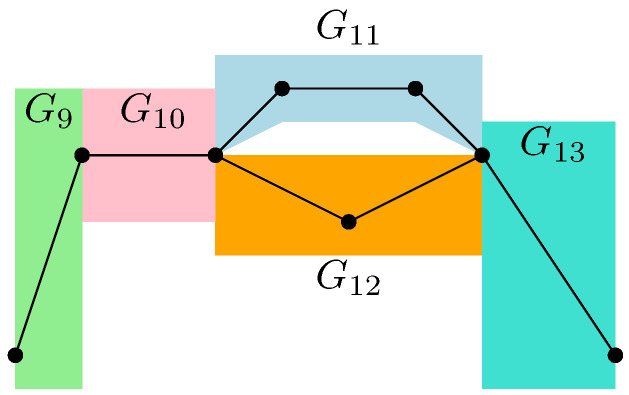
The graph $G_{5}$ factorizes as a series composition of $G_{9}$ and $G_{10}$, with $G_{11}$ composed in parallel to $G_{12}$ and in series with $G_{13}$.

 *The graph* $G_{5}$ *can be decomposed as follows (see Figure 13)*

G5=G9SG10SG11PG12SG13.

 *In terms of the associated probability measures, it is found that*

$$\mu_{G_{5}}=\mu_{G_{9}}⊠\mathrm{MP}⊠\mu_{G_{10}}⊠\mathrm{MP}⊠\left(\mu_{G_{11}}\times \mu_{G_{12}}\right)⊠\mathrm{MP}⊠\mu_{G_{13}}.$$

 *By iteratively applying series compositions, we obtain*

$$\mu_{G_{11}}={\mathrm{MP}}^{⊠2}\;\mathrm{and}\;\mu_{G_{12}}=\mathrm{MP}.$$

*Thus,*

$$\mu_{G_{5}}={\mathrm{MP}}^{⊠3}⊠\left({\mathrm{MP}}^{⊠2}\times \mathrm{MP}\right).$$

*With all of these ingredients, the specified formula for* $\mu_{G_{A|B}^{o}}$ *is obtained.*

**Figure 14:**
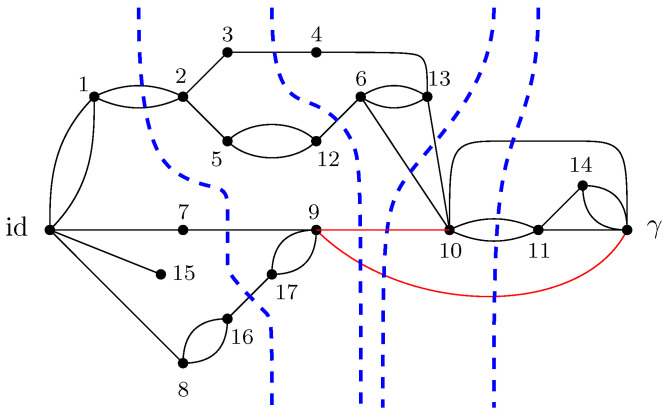
Different cuts of size 4 in the network of Figure 7 are represented with blue dashed lines. The maximum flow in the network is 4. Note that some of these minimal cuts share some edges, which are represented in red.

**Remark** **6.**  *In the example of the tensor network represented in Figure 1, the moments are computed from the factorized series-parallel structure using the flow approach. It should be mentioned that if the minimal cut approach is taken, then minimal intersecting cuts in the network exist, as shown in Figure 14. Therefore, the correction terms of the entropy can be computed as the moment of a given measure without the need for any minimal cut assumptions such as those considered in [3].*

**Remark** **7.**  *The obtained measure $\mu_{G_{A|B}^{o}}$ for a given ordered series-parallel graph $G_{A|B}^{o}$ has compact support where it combines the Marc̆henko–Pastur distribution with the classical product measure and free product convolution constructed from the structure of $G_{A|B}^{o}$.*

## 7. Examples of Series-Parallel Networks

In this section, the results obtained previously are applied to various random tensor networks that possess an induced series-parallel order. The discussion begins with simple cases and progresses towards more physically relevant cases.

### 7.1. Single Vertex Network

**Figure 15:**
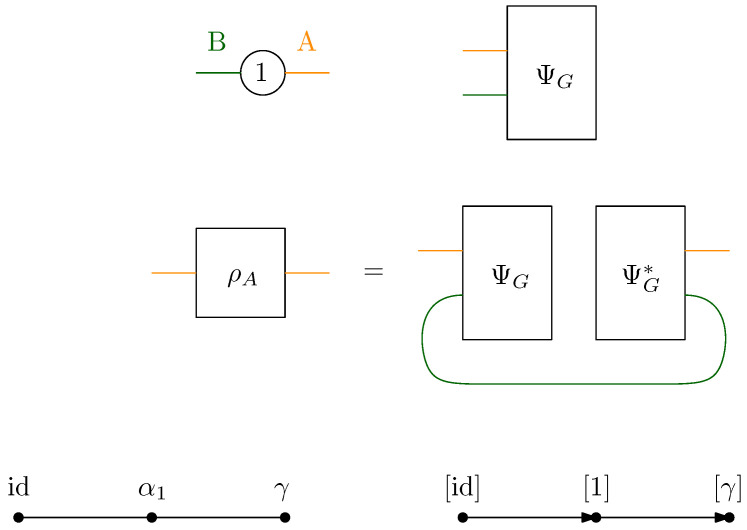
A single vertex network: the top row shows the network and the associated random tensor $\Psi_{G}$, the middle row shows the reduced matrix $\rho_{A}$ obtained by partial tracing of the edge *B* between $\Psi_{G}$ and $\Psi_{G}^{*}$, and the bottom row shows the network $G_{A|B}$ and partial-order graph $G_{A|B}^{o}$.

The simplest possible case is considered first, that is, a tensor network having only one vertex, no bulk edges, and two boundary half-edges; see Figure 15. For this network, the associated random tensor

$$\Psi_{G}\in C^{D}⊗C^{D}$$

has i.i.d. standard complex Gaussian entries. The two boundary half-edges are partitioned into two one-element sets, *A* and $B=\bar{A}$. From this tensor, the reduced matrix is constructed as

$$\rho_{A}:={\mathrm{Tr}}_{B}|\Psi_{G}\rangle \langle \Psi_{G}|$$

by partial tracing of the half-edge *B*. Note that in this very simple case the matrix $\rho_{A}$ can also be seen as a product of the matricization of the tensor $\Psi_{G}$ with its Hermitian adjoint; hence, $\rho_{A}$ is a Wishart random matrix (see Appendix A for the definition and basic properties of Wishart matrices).

To analyze the large *D* spectral properties of $\rho_{A}$, the network $G_{A|B}$ is first constructed by connecting all the half-edges in *G* that belong to *A* to a new vertex γ and connecting those in *B* to a new vertex id. The flow analysis of this network is trivial; a unique path from id to γ exists, meaning that the maximum flow is 1 and the residual network is empty (both edges in the network are used for the construction of the unique maximum flow).

Because there is a unique path achieving maximum flow and only a single vertex in the network, the partial order induced by the path is very simple: $\mathrm{id}-\alpha_{1}-\gamma$. Hence, the only condition on the permutation $\alpha_{1}\in S_{n}$ is that it should lie on the geodesic between the identity permutation id and the full cycle permutation $\gamma \in S_{n}$. Thus, we obtain a series network; see the bottom right-hand diagram in Figure 15. The limit moment distribution is MP, that is, the Marc̆henko–Pastur distribution (of parameter 1). This matches previously obtained results about the *induced measure* of mixed quantum states (density matrices) [39,40,41]. Indeed, the matrix $\rho_{A}$ can be interpreted in quantum information theory as the partial trace of the rank-one matrix in the direction of a random Gaussian vector $\Psi_{G}\in C^{d}⊗C^{d}$. Up to normalization, this random density matrix belongs to the ensemble of induced density matrices. The fact that the two factors of the tensor product have equal dimensions corresponds to taking the *uniform measure* on the (convex, compact) set of density matrices [42,43]. The statistics of the eigenvalues of such random matrices have been extensively studied in the literature. In particular, the asymptotic von Neumann entropy has been studied by Page [44,45,46], who conjectured that

$$ES\left(\rho_{A}\right)=\sum_{i=D+1}^{2D}\frac{1}{i}-\frac{D-1}{2D}∼\log D-\frac{1}{2}\;\;\mathrm{as}\;D\to \infty .$$

The reader is referred to Section 8 for a derivation of such statistics in the context of this work.

### 7.2. Series Network

**Figure 16:**
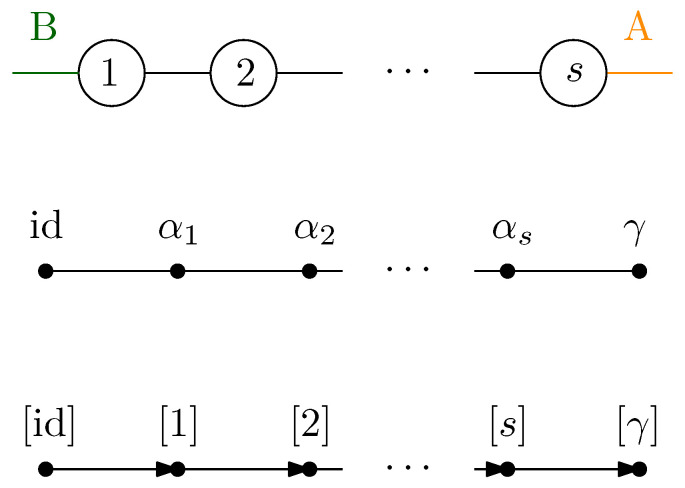
A series network; the *s* vertices of the network are arranged in a line, with two half-edges at the end points. The maximum flow is 1, and is unique. The single path realizing the flow induces a (total) order $\alpha_{1}\leq \alpha_{2}\leq \cdots \leq \alpha_{s}$ on the geodesic permutations.

We now consider a tensor network consisting of *s* vertices arranged in a path graph with two half-edges at the end points. This network is depicted along with the various steps needed to compute the limiting spectrum distribution of the reduced matrix. The network associated with the graph (where the partition of the half-edges is clear) has a single path from the source to the sink, meaning that the maximum flow is unity, see Figure 16.

The residual graph obtained by removing the edges from the unique path achieving maximum flow is empty; hence, the partial order on the vertices is again a total order:

$$\mathrm{id}⪯\alpha_{1}⪯\cdots ⪯\alpha_{s}⪯\gamma .$$

Thus, a *series network* is obtained and the final measure is obtained by applying *s* times the series concatenation procedure from Definition 12, yielding

$$\mu_{G_{A|B}^{o}}=\underset{s\;\mathrm{times}}{\underset{︸}{\mathrm{MP}⊠\mathrm{MP}⊠\ldots ⊠\mathrm{MP}}}={\mathrm{MP}}^{⊠s}.$$

Very similar results were previously obtained by Cécilia Lancien [47]; see also [25]. This measure is commonly known as the *Fuss–Catalan* distribution of order *s* [48]; see also Theorem A5. In combinatorics, its moments are known as the Fuss–Catalan numbers

∫tndMP⊠s(t)=1sn+1sn+nn

and its entropy ([49], Proposition 6.2) is

$$\int -t\log t\;d{\mathrm{MP}}^{⊠s}\left(t\right)=\sum_{i=2}^{s+1}\frac{1}{i}.$$

Such tensor network states have already been considered in quantum information theory [43,49,50].

### 7.3. Two-Dimensional Lattice

**Figure 17:**
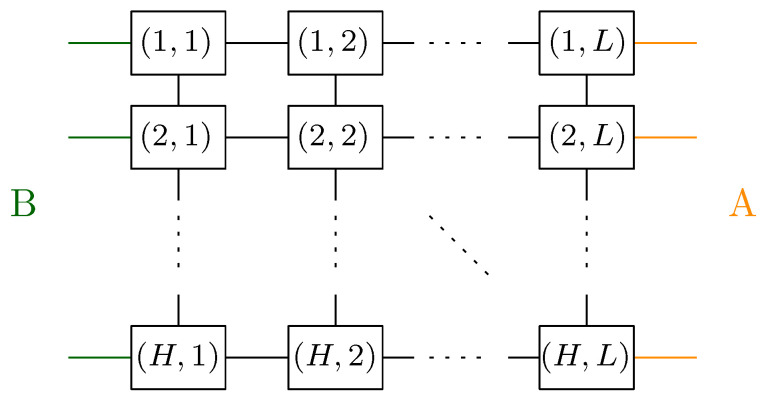
A network corresponding to a H×L 2D lattice. Half-edges are attached to vertices on the left and right boundaries, corresponding to a tensor in ${\left(C^{D}\right)}^{⊗2H}$.

We now discuss a physically relevant network consisting of a rectangle that is part of a 2D lattice (part of $Z^{2}$). Two integer parameters are considered, namely, the length *L* and height *H* of the rectangle, resulting in H·L vertices. The vertices are connected by the edges inherited from the $Z^{2}$ lattice; see Figure 17. The left-most (resp. right-most) columns of vertices are assigned half-edges that belong to the class *B* (resp. A) of the half-edge partition defining the two regions.

**Figure 18:**
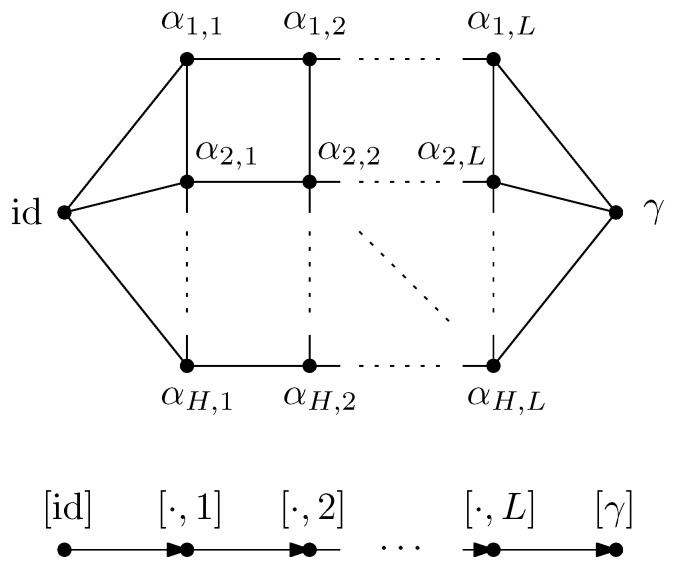
The network associated with the H×L$Z^{2}$ lattice fragment. The maximum flow in this network is *H*, corresponding to horizontal edge-disjoint paths. These *H* paths induce a total order on the *L* vertex clusters, each having *H* vertices.

The flow network corresponding to the graph and the partition A|B is depicted in the top diagram in Figure 18. The maximum flow in this network is *H*; one can consider *H* parallel horizontal paths which go from id to γ. Note that the set of *H* edge-disjoint paths in the network achieving the maximum flow is unique. In this case, the residual network is non-empty, with *H* clusters of the form

$$C_{j}:=\left\{\left[i,j\right]\;:\;i=1,\ldots ,L\right\}.$$

The order relation on the clusters is again a total order on *L* points; see the bottom diagram in Figure 18. Again we obtain the Fuss–Catalan distribution:

$$\mu_{G_{A|B}^{o}}={\mathrm{MP}}^{⊠L}.$$

## 8. Results for Normalized Tensor Network States

This section presents the main technical contribution of this paper. With the help of all the results obtained from the previous sections, the Rényi and von Neumann entropies for a given approximated normalized state ${\tilde{\rho }}_{A}:=D^{-|E_{\partial }|}\rho_{A}$ associated with a given boundary subregion $A\subseteq E_{\partial }$ can be computed. The main results of this section first consist in showing the weak convergence of moments associated with an approximated reduced state ${\tilde{\rho }}_{A}$ that is associated with a given boundary region *A* in Theorem 7. Moreover, Corollary 2 shows the existence of correction terms as moments of a graph-dependent measure, which can be explicitly computed in the case of an obtained series-parallel partial order $G_{A|B}^{o}$.

In Section 8.1, different concentration inequalities will be shown, which in Section 8.2 will allow the main results of this section to be presented.

### 8.1. Concentration

In this subsection, we provide different concentration results which will allow the main technical contribution to be presented in the following subsection.

First, we recall the following theorem which estimates the deviation probability of polynomials in Gaussian random variables. This theorem is relevant for different concentration results that will be proved later in this subsection.

**Theorem** **6**  ([51], Theorem 6.7)**.** *Let g be a polynomial in m variables of degree q; then, if $X_{1},\ldots ,X_{m}$ are independent centered Gaussian random variables,*

$$\forall t>0,\;P\left(|g\left(X_{1},\ldots ,X_{m}\right)-E\;g|>t{\left(\mathrm{Var}\left(g\right)\right)}^{\frac{1}{2}}\right)\leq \exp\left\{\left(-c_{q}\;t^{\frac{2}{q}}\right)\right\},$$

*where* V(g) *is the variance of* $g(X_{1},\ldots ,X_{m})$ *and *$c_{q}$ *is a constant which depends only on q.*

**Proposition** **7.**  *Let G be a bulk connected graph and let* $A\subseteq E_{\partial }$*; then,*

$$P\left(|\mathrm{Tr}{\tilde{\rho }}_{A}-1|>\varepsilon \right)\leq \exp\left\{\left(-c_{\left|E\right|}\varepsilon^{\frac{1}{\left|E\right|}}D^{\frac{|E_{b}|}{2|E|}}\right)\right\},$$

*where* ${\tilde{\rho }}_{A}:=D^{-|E_{\partial }|}\rho_{A}$*.*

**Proof.**  First, it should be noted that $\mathrm{Tr}\rho_{A}$ is a polynomial in $|g_{x}\rangle \in H_{x}$ of total degree 2|E|. Moreover, recall that for a random Gaussian vector $|g_{x}\rangle \in H_{x}$, the following holds:

$$\forall x\in V,\;E\left[|g_{x}\rangle \langle g_{x}|\right]={\mathrm{id}}_{x}\;\mathrm{and}\;E\left[{|g_{x}\rangle \langle g_{x}|}^{⊗2}\right]={\mathrm{id}}_{x}+F_{x}$$

where ${\mathrm{id}}_{x}$ and $F_{x}$ act on all the edges of the Hilbert space to generate the local Hilbert space for each vertex *x*. Moreover, it is implicitly assumed that ${\mathrm{id}}_{x}\equiv {\mathrm{id}}_{x}^{⊗2}$ and that the Swap operator $F_{x}$ is a unitary representation of a permutation element in $S_{2}$.

It is easy to check that the variance $\mathrm{Var}(\mathrm{Tr}{\tilde{\rho }}_{A})$ provides

$$\mathrm{Var}\left(\mathrm{Tr}{\tilde{\rho }}_{A}\right)=E\left[{\left(\mathrm{Tr}{\tilde{\rho }}_{A}\right)}^{2}\right]-{\left(E\left[\mathrm{Tr}({\tilde{\rho }}_{A})\right]\right)}^{2}=O\left(D^{-|E_{b}|}\right),$$

where we have used the fact that

$$\begin{array}{cc} E\left[{\left(\mathrm{Tr}{\tilde{\rho }}_{A}\right)}^{2}\right] & =\mathrm{Tr}\left(\underset{e\in E_{b}}{⨂}|\Omega_{e}\rangle {\langle \Omega_{e}|}^{⊗2}\underset{x\in V}{⨂}E\left[{|g_{x}\rangle \langle g_{x}|}^{⊗2}\right]\right)\\ \; & =1+D^{|E_{\partial }|}\sum_{\alpha_{e}\in S_{2}}\prod_{e\in E_{b}}\mathrm{Tr}\left({\left|\Omega_{e}\rangle \langle \Omega_{e}\right|}^{⊗2}\;U_{\alpha_{e}}\right)\prod_{e\in E_{\partial }}\mathrm{Tr}\left(U_{\alpha_{e}}\right)\\ \; & =1+O\left(D^{-|E_{b}|}\right). \end{array}$$

In the last equality, the bulk contribution is of $D^{-|E_{b}|}$, while the boundary edges contribute with $D^{|E_{\partial }|}$. The second term of the variance is

$$E\left[\mathrm{Tr}({\tilde{\rho }}_{A})\right]=\mathrm{Tr}\left(\underset{e\in E_{b}}{⨂}\left|\Omega_{e}\rangle \langle \Omega_{e}\right|\underset{x\in V}{⨂}E\left[\left|g_{x}\rangle \langle g_{x}\right|\right]\right)=1.$$

By combining the variance $\mathrm{Var}(\mathrm{Tr}\left({\tilde{\rho }}_{A}\right))$ with Proposition 6, we have

$$P\left(|\mathrm{Tr}{\tilde{\rho }}_{A}-E\mathrm{Tr}{\tilde{\rho }}_{A}|>\varepsilon \right)\leq \exp\left\{\left(-c_{\left|E\right|}\varepsilon^{\frac{1}{\left|E\right|}}D^{\frac{|E_{b}|}{2|E|}}\right)\right\},$$

where $\varepsilon :=t\left(D^{-\frac{|E_{b}|}{2}}\right)$ with $c_{\left|E\right|}>0$ is a constant that depends only on the total number of edges |E|. □

**Proposition** **8.**  *Let G be a bulk connected graph and let $A\subseteq E_{\partial }$; then, the following holds:*

$$\forall n>1,\;P\left(|\frac{1}{D^{F(G_{A|B})}}\mathrm{Tr}(\sigma_{A}^{n})-\frac{1}{D^{F(G_{A|B})}}E\left[\mathrm{Tr}\left(\sigma_{A}^{n}\right)\right]|>\varepsilon \right)\leq \exp\left\{\left(-c_{2n|E|}D^{\frac{1}{2n|E|}}\varepsilon^{\frac{1}{n|E|}}\right)\right\},$$

*where $\sigma_{A}:=D^{F(G_{A|B})}{\tilde{\rho }}_{A}$.*

**Proof.**  The proof of this proposition follows the same spirit as the proof of the proposition above. We remark that $\mathrm{Tr}\sigma_{A}^{n}$ is a 2n|E| polynomial in $|g_{x}\rangle$. Moreover the variance was estimated in [33], Lemma 14, where

$$\mathrm{Var}\left(\frac{1}{D^{F(G_{A|B})}}\mathrm{Tr}(\sigma_{A}^{n})\right)=O\left(\frac{1}{D}\right).$$

By defining $\varepsilon :=tD^{-\frac{1}{2}}$, the result follows. □

### 8.2. Entanglement Entropy

This subsection introduces the main technical contribution of this work. With the help of the concentration results, we first assume and use the approximate normalized state ${\tilde{\rho }}_{A}:=D^{-|E_{\partial }|}\rho_{A}$. It is shown that as D→∞, the average Rényi and von Neumann entanglement entropy with correction terms can be computed. In particular, if the obtained partial order is series-parallel, then the correction terms are provided as moments of a partial order-dependent measure $\mu_{G_{A|B}^{o}}$.

Recall from Section 3.2 that the rank of the approximate normalized state is upper-bounded by $D^{F(G_{A|B})}$. We consider the approximate normalized quantum state ${\tilde{\rho }}_{A}$ restricted to its support and its empirical measure $\mu_{A}^{\left(D\right)}$, defined as

$$\sigma_{A}:=D^{F(G_{A|B})}{\tilde{\rho }}_{A}^{S},\;\mathrm{and}\;\mu_{A}^{\left(D\right)}:=\frac{1}{D^{F(G_{A|B})}}\sum_{\lambda \in \mathrm{spec}(\sigma_{A})}\delta_{\lambda },$$

where ${\tilde{\rho }}_{A}^{S}$ is the reduced approximate normalized state restricted on its support. The definition of ${\tilde{\rho }}_{A}$ and the empirical measure $\mu_{A}^{\left(D\right)}$ allow for the demonstration in Theorem 7 of the weak convergence of $\mu_{A}^{\left(D\right)}$ to $\mu_{G_{A|B}}$. In particular, if the obtained partial order $G_{A|B}^{o}$ is series-parallel, then Theorem 5 yields weak convergence to $\mu_{G_{A|B}}$. In Corollary 2, this result allows us to compute the Rényi and von Neumann entanglement entropy.

Recall that a measure $\mu^{\left(D\right)}$*converges weakly* to a measure μ if the following holds for any bounded continuous function f:R→R:

$$\forall \varepsilon >0,\;\lim_{D\to \infty }P\left(\left|\int f\left(t\right)d\mu^{\left(D\right)}\left(t\right)-\int f\left(t\right)d\mu \left(t\right)\right|\leq \varepsilon \right)=1.$$

**Theorem** **7.**  *Let $A\subseteq E_{\partial }$ be a boundary region in the graph G. The empirical measure $\mu_{A}^{\left(D\right)}$ associated to the approximated normalized state $\sigma_{A}$ converges weakly to $\mu_{G_{A|B}}$. More precisely, for all bounded continuous functions f:R→R, we have*

$$\forall \varepsilon >0,\;\lim_{D\to \infty }P\left(\left|\int f\left(t\right)d\mu_{A}^{\left(D\right)}\left(t\right)-\int f\left(t\right)d\mu_{G_{A|B}}\left(t\right)\right|\leq \varepsilon \right)=1.$$

**Proof.**  As shown in Theorem 4, the moments converge to a unique measure $\mu_{G_{A|B}}$. In the particular case of an ordered series-parallel graph $G_{A|B}^{o}$, an explicit graph dependent measure $\mu_{G_{A|B}^{o}}$ is obtained. Recall from Theorem 4 that

$$\frac{1}{D^{F(G_{A|B})}}E[\mathrm{Tr}\left(\sigma_{A}^{n}\right)]\underset{D\to \infty }{\to }m_{n}=\int t^{n}d\mu_{G_{A|B}}\left(t\right).$$

From standard probability theory results, convergence in probability implies weak convergence (see [52], Theorem 25.2). For this, it is only necessary to show the decreasing scaling of the variance as D→∞. Using ([33], Lemma 14), we have

$$\mathrm{Var}\left(\frac{1}{D^{F(G_{A|B})}}\mathrm{Tr}(\sigma_{A}^{n})\right)=O\left(\frac{1}{D}\right),\;\left(D\to \infty \right),$$

establishing the weak convergence of $\mu_{A}^{\left(D\right)}$ to $\mu_{G_{A|B}}$. In particular, if the graph is series-parallel, then we obtain $\mu_{G_{A|B}^{o}}$. □

**Lemma** **1.**  *Let $A\subseteq E_{\partial }$ be a boundary region and let $m_{n}^{\left(D\right)}$ be the moment associated to the empirical measure $\mu_{A}^{\left(D\right)}$. Then, the following holds:*

$$P\left(|E\log\left(m_{n}^{\left(D\right)}\right)-\log\left(Em_{n}^{\left(D\right)}\right)|>\varepsilon \right)\underset{D\to \infty }{\to }1\;\mathrm{where}\;m_{n}^{\left(D\right)}:=\frac{1}{D^{F(G_{A|B})}}E[\mathrm{Tr}\left(\sigma_{A}^{n}\right)].$$

**Proof.**  By Proposition 8 and Jensen’s inequality, $E\log\left(m_{n}^{\left(D\right)}\right)\leq \log\left(Em_{n}^{\left(D\right)}\right)$ holds. It remains to show that $E\log\left(m_{n}^{\left(D\right)}\right)\geq \log\left(Em_{n}^{\left(D\right)}\right)$ holds with high probability. Fixing ε>0, from Proposition 8 it is the case that

$$m_{n}^{\left(D\right)}\geq Em_{n}^{\left(D\right)}-\delta \;\mathrm{with}\;0<\delta \leq \frac{\varepsilon }{\varepsilon +1}Em_{n}^{\left(D\right)}$$

holds with probability $1-\exp\left\{\left(-c_{2n|E|}D^{\frac{1}{2n|E|}}\delta^{\frac{1}{n|E|}}\right)\right\}$. It is easy to check that the following inequalities hold:

$$\begin{array}{cc} \log\left(m_{n}^{\left(D\right)}\right)\geq \log\left(Em_{n}^{\left(D\right)}-\delta \right) & =\log\left(Em_{n}^{\left(D\right)}\right)+\log\left(1-\frac{\delta }{Em_{n}^{\left(D\right)}}\right),\\ \; & \geq \log\left(Em_{n}^{\left(D\right)}\right)-\frac{\delta }{Em_{n}^{\left(D\right)}-\delta }\geq \log\left(Em_{n}^{\left(D\right)}\right)-\varepsilon . \end{array}$$

Therefore, $E\log\left(m_{n}^{\left(D\right)}\right)\geq \log\left(Em_{n}^{\left(D\right)}\right)-\varepsilon$ occurs with probability at least

$$1-\exp\left\{\left(-c_{2n|E|}D^{\frac{1}{2n|E|}}\delta_{\max}^{\frac{1}{n|E|}}\right)\right\}\;\mathrm{where}\;\delta_{\max}=\frac{\varepsilon }{\varepsilon +1}Em_{n}^{\left(D\right)}.$$

As D→∞, $Em_{n}^{\left(D\right)}$ converges; hence, $\delta_{\max}=O\left(1\right)$, showing that the probability estimate above converges to 1 and completing the proof. □

For completeness, the following proposition from [30] is recalled, which plays a key role in the proof of the main result.

**Proposition** **9**  ([30], Proposition 4.4)**.** *Let f be a continuous function on R with polynomial growth and with $\nu_{n}$ being a sequence of probability measures that converges in moments to a compactly supported measure ν. Then, $\int fd\nu_{n}\to \int fd\nu$.*

**Corollary** **2.**  *Let $A\subseteq E_{\partial }$ be a boundary region in G and let ${\tilde{\rho }}_{A}$ be the approximated reduced normalized state. Then, the averaged Rényi and von Neumann entropies converge weakly as D→∞, and the following holds:*

$$\begin{array}{cc} F\left(G_{A|B}\right)\log D-ES_{n}\left({\tilde{\rho }}_{A}\right) & \underset{D\to \infty }{\to }\frac{1}{n-1}\log\left(\int t^{n}\;d\mu_{G_{A|B}}\right),\\ F\left(G_{A|B}\right)\log D-ES\left({\tilde{\rho }}_{A}\right) & \underset{D\to \infty }{\to }\int t\;\log t\;d\mu_{G_{A|B}} \end{array}$$

*where $F\left(G_{A|B}\right):=\mathrm{maxflow}\left(G_{A|B}\right)$.*

**Proof.**  The proof of this corollary is a direct consequence of the concentration results obtained in the previous subsection and the weak convergence of $\mu_{A}^{\left(D\right)}$ to $\mu_{G_{A|B}}$.

First, the Rényi entropy is considered. For this, we have

$$\begin{array}{c} F\left(G_{A|B}\right)\log D-ES_{n}\left({\tilde{\rho }}_{A}\right)=\frac{1}{1-n}E\log\left(m_{n,A}^{\left(D\right)}\right),\;\mathrm{where}\;m_{n}^{\left(D\right)}:=\frac{1}{D^{F(G_{A|B})}}E[\mathrm{Tr}\left({\left(\sigma_{A}\right)}^{n}\right)] \end{array}$$

and recall that $\sigma_{A}:=D^{F(G_{A|B})}{\tilde{\rho }}_{A}^{S}$ is restricted to the support of ${\tilde{\rho }}_{A}:=D^{-|E_{\partial }|}\rho_{A}$. Using Lemma 1 and in the limit D→∞, we have

$$F\left(G_{A|B}\right)\log D-ES_{n}\left({\tilde{\rho }}_{A}\right)\underset{D\to \infty }{\to }\frac{1}{n-1}\log\left(\int t^{n}\;d\mu_{G_{A|B}}\right).$$

For the von Neumann entropy, let $\left\{\lambda_{i}\right\}\in \mathrm{spec}\left(\sigma_{A}\right)$ and $\left\{{\tilde{\lambda }}_{i}\right\}\in \mathrm{spec}\left({\tilde{\rho }}_{A}\right)$. It follows directly that

$$ES\left({\tilde{\rho }}_{A}\right)=-E\sum_{i}{\tilde{\lambda }}_{i}\log\left({\tilde{\lambda }}_{i}\right)=F\left(G_{A|B}\right)\log\left(D\right)-\frac{1}{D^{F(G_{A|B})}}E\sum_{i}\lambda_{i}\log\left(\lambda_{i}\right).$$

Defining the function f:R→R as f(t):=tlogt, by combining Proposition 9 and Theorem 7 we obtain the following weak convergence as D→∞:

$$F\left(G_{A|B}\right)\log D-ES\left({\tilde{\rho }}_{A}\right)=\frac{1}{D^{F(G_{A|B})}}\;E\left(\sum_{i}\;f\left(\lambda_{i}\right)\right)\underset{D\to \infty }{\to }\int \;f\left(t\right)\;d\mu_{G_{A|B}}$$

where the measure $\mu_{G_{A|B}}$ is defined on a compact support, completing the proof of the corollary. In the particular case where the obtained poset structure $G_{A|B}^{o}$ is series-parallel, the obtained graph dependent measure is explicitly provided by $\mu_{G_{A|B}}=\mu_{G_{A|B}^{o}}$ using Theorem 5. □

## 9. Conclusions

Given a general graph with a boundary region and a bulk region, the main goal of this work is to compute the entanglement entropy (the Rényi and the von Neumann entropy) of a given sub-boundary region *A* of the graph. By analyzing the moments of a state associated with the region *A* as D→∞ and assisted by the (maximal) flow approach, we compute the leading terms contributing to the moment. By analyzing and removing all of the augmenting paths of the network $G_{A|B}$ constructed by connecting the region *A* to the total cycle γ and id to the region *B* starting from id and ending in γ, a cluster graph $G_{A|B}^{c}$ is obtained by identifying all of the remaining edge-connected permutations. The flow approach induces a natural-order poset structure, represented by the induced poset order $G_{A|B}^{o}$. The maximal flow approach allows for the deduction of the moment convergence to the moment of a unique graph-dependent measure $\mu_{G_{A|B}}$. This result allows for the deduction of the higher-order correction terms of the Rényi and von Neumann entropy, provided by a graph-dependent measure $\mu_{G_{A|B}}$. Moreover, it has been shown that with the help of free probability theory, if the obtained partial order $G_{A|B}^{o}$ is series-parallel, then the associated graph-dependent measure $\mu_{G_{A|B}}=\mu_{G_{A|B}^{o}}$ can be explicitly provided, which contributes to the higher-order correction terms of each of the Rényi and von Neumann entanglement entropies.

In this work, no assumption regarding the minimal cuts is made; from the duality in the maximal flow approach, different minimal cuts can be obtained which may intersect in different edges. Moreover, the higher-order correction terms in the entanglement entropy can describe the quantum corrections beyond the area law behavior of the expected Ryu–Takayanagi entanglement entropy in the context of AdS/CFT. It has been previously argued in the literature that if higher-order correction terms in the random tensor network setting are to be considered, it is necessary to go beyond the maximally entangled state and consider general link states representing the bulk matter field. In this work, the obtained higher-order quantum fluctuation of entanglement entropy is achieved with only maximally entangled states, which we interpret as fluctuations of spacetime itself without any need for bulk fields represented by a generic link state.

## Data Availability

No data has been generated or used in this work.

## Appendix A. Basics of the Combinatorial Approach to Free Probability Theory

In this section, the necessary material on *combinatorics* and *free probability* needed to understand the rest of this section is recalled and presented. All of the material introduced here is standard and can be found in [53,54].

Let $\pi :=\{V_{1},\cdots ,V_{n}\}$ (not to be confused with $\pi_{i}$ introduced in Section 5 representing the different paths) be a partition of a finite totally-ordered set *S* such that ${⨆}_{i\in [n]}V_{i}=S$. The sets $\left\{V_{i}\right\}$ are called the *blocks* of π. The notation $p{∼}_{\pi }q$ is used if *p* and *q* belong to the same block of π. A partition π of a set *S* is called *crossing* if there exist $p_{1}<q_{1}<p_{2}<q_{2}$ in *S* such that $p_{1}{∼}_{\pi }p_{2}{≁}_{\pi }q_{1}∼q_{2}$. A partition is called *non-crossing* if π does not cross. The set of non-crossing partitions of *S* is denoted by NC(S). In particular, if S={1,⋯n}, then the non-crossing partition set is denoted by NC(n). The set of non-crossing partitions plays a crucial role in various areas, from combinatorics [55] to random matrices and free probability theory, which is the main focus here. Moreover, the following crucial result is noted [53]: *there exists a one-to-one correspondence between the non-crossing partition set and the set of permutations α in a geodesic between γ and* id, *i.e., $\left|\alpha |+\right|\alpha^{-1}\gamma |=|\gamma |$.* Another important fact is that the cardinality $\left|\mathrm{NC}\left(n\right)\right|={\mathrm{Cat}}_{n}$, where

(A1) Catn:=1n+12nn

are the *Catalan numbers*. For more combinatorial details and properties of Catalan numbers and non-crossing partitions, see [53]. We assume that $\left(\alpha_{1},\cdots ,\alpha_{k}\right)$ is a *k*-tuple of permutations in $S_{n}$ such that

(A2) $$|\alpha_{1}|+\sum_{i\in [k-1]}|\alpha_{i}^{-1}\alpha_{i+1}\left|+\right|\alpha_{k}^{-1}\gamma |=|\gamma |.$$

These are geodesics between id and γ. The cardinality of the set of *k*-tuple permutations satisfying the geodesic Equation (A2) is known as the *Fuss–Catalan* numbers, provided by

FCn,k:=1nk+1n+nkn,

which generalize the Catalan numbers for k=1.

The *free probability theory* tools used in this work are now introduced. The intrinsic link between free probability theory and combinatorics is also noted, and some examples are provided to illustrate it. The combinatorics presented here will be used in the rest of this section to help understand the main result.

A *non-commutative probability space* is defined as a pair (A,ω), where A is a unital $C^{*}$-algebra with a state ω:A→C such that $\omega (1_{A})=1$. The elements a∈A are called *non-commutative variables*. In the non-commutative probability space, the distribution law $\mu_{a}$ can be associated to a∈A, defined as $\mu_{a}=\omega \left(a\right)$.

Before providing concrete examples of non-commutative probability spaces, the notion of *freeness*, which plays a crucial role in the non-commutative probability world, is recalled. The notion of freeness generalizes “classical” independence when the algebra A is commutative. For *n* given non-commutative random variables $\{a_{i}\}\in A$, these are said to be *free independent* if the following holds for any polynomials $\left\{p_{i}\right\}$:

(A3) $$\omega (a_{1}a_{2}\cdots a_{n})=0$$

whenever $\omega (p_{k}\left(a_{i_{k}}\right))=0$ for k∈[n] and for two non-adjacent indices $i_{k}$ and $i_{k+1}$. With the definition of free independence, the following can be checked for two free independent variables $a_{1}$ and $a_{2}$:

(A4) $$\omega (\left(a_{1}-\omega \left(a_{1}\right)\right)\left(a_{2}-\omega \left(a_{2}\right)\right)=\omega \left(a_{1}a_{2}\right)-\omega \left(a_{1}\right)\omega \left(a_{2}\right)=0$$

which generalizes the notion of standard independence in the commutative setting, where $E\left(a_{1}a_{2}\right)=E\left(a_{1}\right)E\left(a_{2}\right)$ for two commutative random variables $a_{1},a_{2}$ in a commutative probability space.

**Definition** **A1.** 
*Let $\left(A_{N},\omega_{N}\right)$ with N∈N and (A,ω) be non-commutative probability spaces. The sequence $a_{N}\in A_{N}$ is said to converge weakly to a∈A as N→∞ if the following holds:*

(A5)
$$\lim_{N\to \infty }\omega_{N}\left({\left(a_{N}\right)}^{n}\right)=\omega \left(a^{n}\right)\;\forall n\in N,$$

*where $\omega \left(a^{n}\right)=\int x^{n}d\mu_{a}\left(x\right)$ are the moments of a.*

To illustrate concrete non-commutative probability spaces, some classical examples are provided. The first example we consider is the “classical” probability space corresponding to a commutative algebra. Let (Ω,Σ,μ), where Ω is a set, Σ is a σ-algebra, and μ is a probability measure. We define $A:=L^{\infty -}\left(\Omega ,\mu \right)$, where

$$L^{\infty -}\left(\Omega ,\mu \right):=\underset{1\leq k<\infty }{⋂}L^{k}\left(\Omega ,\mu \right),$$

and define the state ω as

$$\omega \left(a\right):=\int_{\Omega }a\left(x\right)d\mu \left(x\right),\;a\in A.$$

The tuple (A,ω) defines a commutative probability space. Another standard example is the random matrices case. The algebra A consisting of k×k matrices over $L^{\infty -}\left(\Omega ,\mu \right)$ is considered, where

$$A:=M_{k}\left(L^{\infty -}\left(\Omega ,\mu \right)\right).$$

The state ω on A is defined as

$$\omega \left(a\right):=\int_{\Omega }tr\left(a\left(x\right)\right)d\mu \left(x\right),\;a\in A,$$

where tr(·) is the normalized trace. The space (A,ω) defines a non-commutative probability space, namely, the space of random matrices over (Ω,Σ,μ).

For a given non-commutative random variable a∈A, the *n*th moments of *a* are provided by

(A6) $$m_{n}\left(\mu_{a}\right):=\int t^{n}d\mu_{a}\left(t\right).$$

Moreover, for given random variables $\left\{a_{1},\cdots ,a_{n}\right\}$ in A, the moments are provided by

(A7) $$\omega \left(a_{1}\cdots a_{n}\right):=\sum_{\pi \in \mathrm{NC}(n)}\kappa_{\pi }\left(a_{1},\cdots ,a_{n}\right),$$

where $\kappa_{\pi }$ are the *free cumulants*. The above equation is known as the *moments–cumulants formula*, where the free independence can be characterized by the vanishing of mixed cumulants (see [53], Chapter 11).

In free probability theory, a “convolution operation” can be defined for two free independent random variables $a_{1},a_{2}\in A$. In most of this work, only the *free multiplicative convolution* is considered. Let $a_{1}$ and $a_{2}$ be two free independent random variables in A with their respective distributions $\mu_{a_{1}}$ and $\mu_{a_{2}}$; it is assumed in what follows that $\mu_{a_{1}}$ and $\mu_{a_{2}}$ are compactly supported and that their support is a subset of $R_{+}$. A free multiplicative convolution, or simply a *free product*, is defined by

(A8) $$\mu_{a_{1}a_{2}}:=\mu_{a_{1}}⊠\mu_{a_{2}},$$

where $\mu_{a_{1}a_{2}}$ represents the distribution of $a_{1}\;a_{2}$. As for the free additive convolution, there exists a standard and analytical way to compute the free product, which can be done via the *S-transform*. The *S-transform* of a probability distribution $\mu_{a}$ is defined as *the formal inverse of the moment-generating formal power series*, provided by

(A9) $$S_{\mu_{a}}\left(z\right)=\frac{1-z}{z}M_{a}^{-1}\left(z\right),$$

where $M_{a}^{-1}\left(z\right)$ is the formal inverse of the *moment-generating* formal power series provided by

(A10) $$M_{\mu_{a}}\left(z\right):=\sum_{k=1}^{\infty }\;m_{k,a}\;z^{k}.$$

With the help of the S-transform, the free product can be computed, where:

(A11) $$S_{\mu_{a_{1}^{1/2}a_{2}a_{1}^{1/2}}}\left(z\right)=S_{\mu_{a_{1}}}\left(z\right)\;S_{\mu_{a_{2}}}\left(z\right).$$

In the following, we recall some standard distributions that are well-known in the literature and highly used in this work.

The first distribution considered here is the *semicircular law*, which is one of the most important distributions encountered in free probability theory. The semicircular distribution $\mu_{\mathrm{SC}}\left(x\right)$ is defined by the density:

(A12) $$d\mu_{\mathrm{SC}}\left(x\right):=\frac{\sqrt{4-x^{2}}}{2\pi }1_{x\in [-2,2]}dx.$$

For illustration, the S-transform of the semicircular distribution can be computed by standard computation:

$$S_{\mu_{\mathrm{SC}}}\left(z\right)=\frac{-z+\sqrt{z^{2}-4}}{2}.$$

The first link that can be identified is that the moments of the semicircular distribution are intrinsically related to the Catalan numbers. The following equality holds:

(A13) $$\int x^{k}d\mu_{\mathrm{SC}}\left(x\right)={\mathrm{Cat}}_{k}$$

where ${\mathrm{Cat}}_{k}$ are the Catalan numbers (see Equation (A1) and Chapter 2 in [53] for more details). Moreover, the S-transform provides another important link between free probability and combinatorics by allowing the computation of the moments of free product convolution of Marc̆henko–Pastur distribution (see Theorem A5).

Another well-known result due to Wigner [56] shows the following link between the semicircular distribution and random Gaussian matrices.

**Theorem** **A1** ([56])**.** *Let N∈N and let $A_{N}$ be an N×N self-adjoint random Gaussian matrix. Then, $A_{N}$ converges weakly to a semicircular distribution $\mu_{SC}\left(x\right)$.*

A complete proof can be found in [57]. In this particular case, a deep link is established between random Gaussian matrices, the semicircular law, and the Catalan numbers. Moreover, the semicircular law plays an important role in free probability theory as a free central limit distribution. Next, one of the main results in free probability theory is recalled (see Theorem 8.10 in [53]).

**Theorem** **A2.** 
*Let (A,ω) be a non-commutative probability space and let $a_{1},\cdots ,a_{N}\in A$ be free independent and identically distributed self-adjoint random variables. Assume that $\omega (a_{i})=0$ for i∈[N] and denote the variance of the random variables $a_{i}$ by $\sigma^{2}:=\omega \left(a_{i}^{2}\right)$. Then, the following holds:*

$$\frac{a_{1}+\cdots +a_{N}}{\sqrt{N}}\to \mu_{SC}\left(x\right).$$

*The sequence converges weakly to $\mu_{SC}\left(x\right)$ as N→∞, where s is a semicircular variable of variance $\sigma^{2}$.*

This particular distribution helps to illustrate the relationship between random matrices, combinatorics, and free probability theory.

In the following, we provide another example of a distribution that plays an important role in this work. The second distribution considered here is the *Marc̆henko–Pastur distribution*, denoted by MP(t) and defined by

$$\begin{array}{cc} \mathrm{MP}(t) & :=\max\left(1-t,0\right)\delta_{0}+\nu_{t},\\ d\nu_{t}\left(x\right) & :=\frac{\sqrt{4t-{\left(x-1-t\right)}^{2}}}{2\pi x}1_{{\left(x-1-t\right)}^{2}\leq 4t}dx. \end{array}$$

The Marc̆henko–Pastur distribution is deeply related to Wishart matrices. Let *Z* be a Wishart matrix defined as $Z:=\frac{1}{m}YY^{*}$, where $Z\in M_{nm}\left(C\right)$ and the entries of $Y\in M_{nm}\left(C\right)$ are complex random Gaussian variables. It was shown by Marc̆henko and Pastur that the empirical distribution of Wishart matrices converges to the MP(t) defined above. More precisely, the following theorem was established.

**Theorem** **A3.** 
*Consider a Wishart matrix Z and let $\mu_{n,m}$ be its empirical distribution provided by*

$$\mu_{n,m}:=\frac{1}{n}\sum_{z\in \mathrm{spec}(Z)}\delta_{z}.$$

*Assuming that n/m converges to t as n→∞, then $\mu_{n,m}$ converges (weakly) to MP(t) with t>0.*

For a proof and a more detailed statement of this result, see Theorem 3.6 and Theorem 3.7 in [58]. In particular, the case of MP is of importance in this work, where the distribution is as follows:

(A14) $$d\mathrm{MP}:=\frac{1}{2\pi }\sqrt{4x^{-1}-1}\;dx$$

where the shorthand notation MP is used instead of MP(1).

For the semicircular distribution described previously, the moments of free convolution products of MP can be related to the Fuss–Catalan numbers. For conciseness, only some relevant results for the Marc̆henko–Pastur distribution are stated here; for more details and proofs, see [48].

**Theorem** **A4.** 
*Let MP(t) be the Marc̆henko–Pastur distribution. The S-transform is provided by*

$$S_{\mathrm{MP}(t)}\left(z\right)=\frac{1}{t+z}.$$

One of the main results of [48] was the relation between the free product convolution of MP(t) and combinatorics. Here, we state only the particular result for MP that is relevant for this work.

**Theorem** **A5.** 
*Let MP denote the Marc̆henko–Pastur distribution. For ${\mathrm{MP}}^{⊠s}$ with s≥2, the following holds:*

(A15)
$$\int_{R}x^{n}d{\mathrm{MP}}^{⊠s}={\mathrm{FC}}_{n,s}$$

*where ${\mathrm{FC}}_{n,s}$ are the Fuss–Catalan numbers.*
